# The robotic era: 11-year retrospective study of cholecystectomies at a veterans affairs hospital

**DOI:** 10.1007/s00464-025-12185-3

**Published:** 2025-09-04

**Authors:** Tess C. Huy, Kasey Fitzsimmons, Joon Park, Mark Sawicki, Jeffrey L. Sebastian, James S. Tomlinson, Mark D. Girgis MD

**Affiliations:** 1 Veterans Health Administration, Greater Los Angeles Healthcare System, Los Angeles, CA USA; 2 Department of Surgery, UCLA David Geffen School of Medicine, 10833 Le Conte Ave 54-117 CHS, Los Angeles, CA 90095-6904 USA

**Keywords:** Robotic cholecystectomy, Robotic acute care surgery, Minimally invasive acute care surgery, Acute cholecystitis, Robotic cholecystectomy conversion, Veterans Affairs Hospital

## Abstract

**Background:**

Robotic surgery has been proposed as an approach to mitigate open surgery, which is associated with increased morbidity and worse outcomes when compared to minimally invasive cholecystectomies. The study objective was to determine the effect on conversion rates and outcomes following the adoption of robotic surgery for benign gallbladder disease in a high-risk population.

**Methods:**

Patients ≥ 18 years of age who underwent cholecystectomy for benign gallbladder disease from January 1, 2013 to April 18, 2025 at a Veterans Affairs hospital were retrospectively identified. Primary outcome was rate of conversion to open surgery. Secondary outcomes included post-operative complications and 30-day re-admissions and emergency department visits. Outcomes were compared between surgical approach eras and between robotic and non-robotic cohorts. Univariate and multivariate analysis were performed adjusting for patient factors, surgical factors, and diagnosis.

**Results:**

636 patients (median [IQR], 61 [46, 70] years; 86.0% male) underwent a cholecystectomy most commonly for acute cholecystitis (33.2% of surgical indications). 34.4% of patients underwent surgery during the pre-robotic era, 39.2% during the transition era, and 26.4% during the robotic era. Conversion rates decreased over time (14.6% pre-robotic, 4.0% transition, and 0.0% robotic era; *p* <  0.001). No conversions occurred during robotic cholecystectomy. Odds ratios of composite post-operative complications, 30-day readmissions, and 30-day emergency department visits by era were similar.

**Conclusions:**

Following adoption of robotic cholecystectomy for benign gallbladder disease, conversion to open and primary open surgery were safely eradicated. Use of robotic surgery for patients at highest risk for conversion or with severe disease should be considered.

**Supplementary Information:**

The online version contains supplementary material available at 10.1007/s00464-025-12185-3.

Laparoscopic cholecystectomy is the standard of care compared to open surgery for benign gallbladder disease due to decreased length of stay, improved post-operative pain, and quality of life [1, 2]. Moreover, when performed for acute cholecystitis, laparoscopic cholecystectomy is associated with much less morbidity [3, 4]. As expected, when conversion from laparoscopic to open cholecystectomy does occur, it is associated with increased rates of surgical site infections and sepsis, 30-day morbidity, and length of stay [5, 6]. While the frequency of open cholecystectomies has continued to decline, conversion to open cholecystectomy remains a persistent risk of laparoscopic surgery [7]. Reported rates of laparoscopic to open conversion range between 0.2 and 9% [6, 8–10]. However, in high-risk male or elderly populations [9, 11–13] and those with certain pre-operative conditions, such as gallbladder disease requiring a percutaneous cholecystostomy tube, conversion rates can be nearly 40% [14, 15]. Within veteran-specific studies containing a largely male and older population, the rate is approximately 9% [6]. In addition, this high risk population is often subjected to open surgery at a rate as high as 14%, without an attempt at laparoscopy [16]. Extrapolating from this data, up to 23% of Veteran Affairs (VA) patients are at risk of an open cholecystectomy and its concomitant morbidity.

Although the decision to start or convert to open surgery traditionally serves as a technique to ensure the completion of a safe cholecystectomy, patients have worse outcomes, nonetheless. Few advances in technology have improved these open surgery or conversion rates. However, in the 2010s, evidence emerged demonstrating superior cholecystectomy conversion rates using a robotic approach with otherwise equivalent safety outcomes [9, 17]. Recognizing that a robotic cholecystectomy approach may reduce conversion rates and subsequent complications, a decision was made to transition to robotic cholecystectomies within the high-risk population at the Greater Los Angeles (GLA) VA hospital.

In 2016, the first robotic cholecystectomy was performed at the GLA VA hospital. By the end of 2021 all cholecystectomies for benign pathology were performed robotically. In this study, we hypothesized that the robotic platform could be harnessed to minimize the opportunity for open surgery whilst maintaining optimal surgical safety in the treatment of benign gallbladder disease.

## Materials and methods

### Study population and design

Patients age ≥ 18 years old who underwent a cholecystectomy for benign gallbladder disease at the GLA VA hospital from January 1, 2013 through April 18, 2025 were retrospectively identified using Veterans Health Information Systems and Technology Architecture (VISTA) surgical case records. Patients undergoing a cholecystectomy via all techniques (laparoscopic, open, or robotic) were included. Cholecystectomies performed for cancer, with cancer on final pathology, or performed in conjunction with a major secondary surgery (e.g. ventral hernia repair) were excluded (n = 15). Primary repair of umbilical hernias at a port site were not considered a secondary surgery and thus were included.

Patient demographic, medical, and laboratory admission data, pathologic data, surgical (intra-operative and post-operative) data, and follow-up data were extracted from the VA Computerized Patient Record System (CPRS) through chart review. Operative and post-operative data were obtained from review of individual operative notes and nursing records. Estimated blood loss was obtained from the surgeon’s record, or nursing records, if unavailable in the operative note.

This study was reviewed and approved by the Institutional Review Board (IRB 1765104-1).

### Paradigm shift

In 2016, the first robotic cholecystectomy was performed at the GLA VA hospital. Since that time, a concerted effort was made to apply the robotic platform for cholecystectomies performed for benign gallbladder disease, including gallstone pancreatitis, choledocholithiasis, gallbladder polyps, etc. The timeframe from 2013 until the first robotic cholecystectomy was performed in 2016 is referred to as the pre-robotic era (01/01/2013–12/01/2016). The timeframe during which the robotic platform was utilized selectively is referred to as the transition era (12/02/2016–11/17/2021). The period during which the robotic platform was utilized exclusively is referred to as the robotic era (11/18/2021–04/18/2025).

The da Vinci Xi was utilized for all surgeries. During the robotic era, all general surgeons (four) at the GLA VA hospital were trained to use the robotic platform. Nine surgeons performed cholecystectomies during the study period, with one surgeon joining the practice in 2018 and with the same four surgeons performing all cases from 2020 through the end of the study period. Only one of the robotic surgeons was considered expert, while the remaining three had performed fewer than ten robotic surgeries and thus considered novice. The GLA VA is an academic institution with participating surgical resident physicians. Though designated robotic physician assistants are employed by the GLA VA, none were involved in the cholecystectomy surgeries. No patients with gallbladder disease were transferred to outside facilities secondary to complexity or severity of disease. Of note, typical practice pattern for patients with choledocholithiasis at the GLA VA is endoscopic retrograde cholangiopancreatography (ERCP) followed by cholecystectomy rather than intra-operative cholangiogram.

### Outcomes

Patient, laboratory, surgical, and peri-operative data were compared between the three eras (pre-robotic, transition, and robotic eras). The primary outcome of interest was rates of conversion from minimally invasive to open surgery. Secondary outcomes of interest included post-operative complications, re-admission 30 days after discharge, and emergency department visits 30 days after discharge.

Secondary analysis of these outcomes was performed between the robotic surgery cohort and the composite of non-robotic surgery patients (the cohort of patients undergoing either laparoscopic cholecystectomy or primary open cholecystectomy as well as those undergoing laparoscopic conversion to open), referred to as the “non-robotic” cohort. Subgroup analyses were performed for patients with a pre-operative surgical indication of acute cholecystitis and with percutaneous cholecystostomy tubes present at the time of surgery.

### Statistical analysis

All relevant patient, laboratory, pathologic, surgical, and post-operative variables were included in univariate analysis. Chi-squared and Fischer’s exact tests were used to analyze categorical data. Wilcoxon rank sum and Kruskal–Wallis tests were used to analyze non-parametric data. T-tests and ANOVA were used to analyze parametric data. Multivariable logistic regression models were used to analyze complications, re-admissions, and emergency department presentations; clinically relevant variables were included in multivariable analysis. Variables that demonstrated collinearity were excluded. All analyses were performed using Stata/SE, version 18.0. Statistical significance was set at 2-sided *p* < 0 0.05. Data were analyzed from May 29 to July 05, 2025.

## Results

### Surgical trends and paradigm shift

**Fig. 1:**
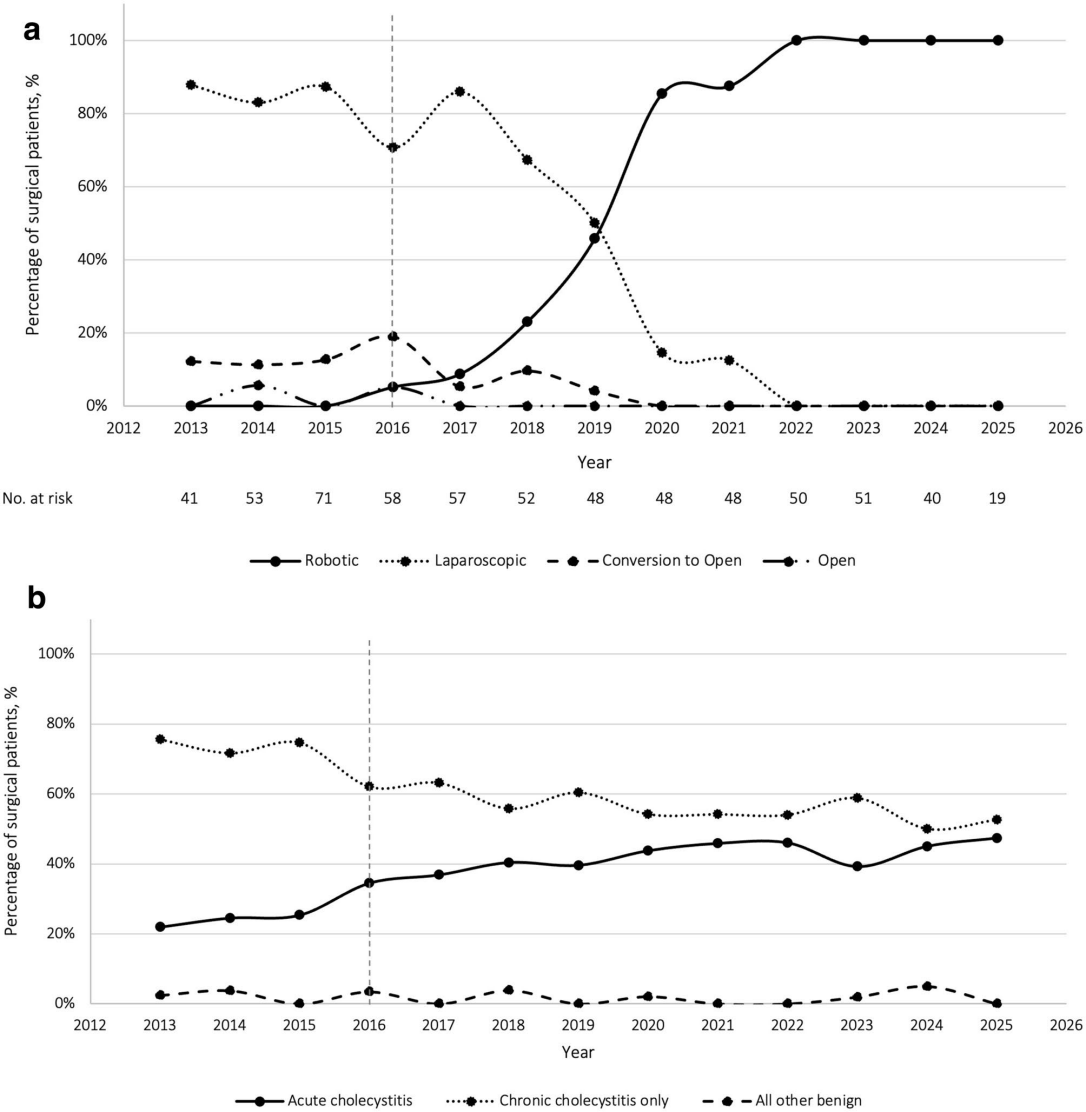
Trends over time (2013–2025) of cholecystectomy surgical technique (**a**) and final pathology (**b**). Vertical dashed line represents first robotic cholecystectomy performed at the Greater Los Angeles Veterans Affairs hospital in 2016

We identified 636 patients who underwent a cholecystectomy at the GLA VA hospital between 2013 and 2025 who met inclusion criteria. By surgical technique, laparoscopic cholecystectomy, robotic cholecystectomy, laparoscopic converted to open, and primary open cholecystectomy constituted 304 (47.8%), 285 (44.8%), 41 (6.9%), and 6 (0.9%) of the total cohort, respectively (Table 2). In 2016, the first robotic cholecystectomy was completed with a total of three (5.2%) robotic cholecystectomies performed that year. By 2020, 85.4% of cholecystectomies were performed robotically and by 2022, 100% were performed robotically (Fig. 1).

### Comparison of eras as defined by the application of the robotic platform

Of the 636 patients included in this study, 219 patients (34.4%) underwent cholecystectomies during the pre-robotic era, 249 patients (39.2%) underwent cholecystectomies during the transition era, and 168 patients (26.4%) underwent a robotic cholecystectomy during the robotic era. The median age at the time of surgery was 61 years (IQR 46, 70) and non-significant between the three eras. Most patients were male (86.0%), white (64.8%), and not Hispanic or Latino (66.0%). Acute cholecystitis was the most common surgical indication (n = 211, 33.2%). Chronic cholecystitis accounted for 20.9% (n = 133) of the total cohort.

In the robotic era more patients had an American Society of Anesthesiologists (ASA) classification of 3 or 4 (76.0% in the robotic era vs 65.6% in the pre-robotic era and 62.6% in the transition era, *p* = 0.041), an arrhythmia (14.9% vs 8.2% and 7.6%, *p* = 0.032), and respiratory diagnosis (50.6% vs 32.0% and 34.1%, *p* <  0.001). Compared to patients in the pre-robotic and transition eras, patients in the robotic era had higher frequency of previous abdominal surgery (*p* = 0.091). Surgical indication was significantly different between eras with acute cholecystitis accounting for the largest proportion of patients (36.3% in the robotic era vs 23.3% in the pre-robotic era and 39.8% in the transition era, *p* = 0.001; Table 1). Complete demographic and clinical characteristics in Supplemental Table 1.

**Table 1:** Demographic and clinical characteristics of patients

Characteristics	Total (%)	Pre-robotic era (%)	Transition era (%)	Robotic era (%)	*p* value
	636 (100.0)	219 (34.4)	249 (39.2)	168 (26.4)	
***Patient characteristics***					
Age, median [IQR]	61 [46, 70]	60 [46, 67]	62 [44, 70]	61 [47, 74]	0.195
Sex, male	547 (86.0)	194 (88.6)	205 (82.3)	148 (88.1)	0.099
Race					0.503
White	412 (64.8)	147 (67.1)	155 (62.2)	110 (65.5)	
Black or African American	115 (18.1)	43 (19.6)	45 (18.1)	27 (16.1)	
Asian	249 (4.6)	7 (3.2)	11 (4.4)	11 (6.6)	
American Indian or Alaskan Native	3 (0.5)	2 (0.9)	0 (0.0)	1 (0.6)	
Native Hawaiian or other Pacific Islander	9 (1.4)	2 (0.9)	4 (1.6)	3 (1.8)	
Two or more races	4 (0.6)	1 (0.5)	3 (1.2)	0 (0.0)	
Declined or unknown or unanswered	64 (10.1)	17 (7.8)	31 (12.4)	16 (9.5)	
History of prior surgery					0.091
Abdominal	204 (32.1)	75 (34.2)	66 (26.5)	63 (37.5)	
Non-abdominal or none	427 (67.1)	143 (65.3)	181 (72.7)	103 (61.3)	
Unknown	5 (0.8)	1 (0.5)	2 (0.8)	2 (1.2)	
ASA (n = 634)^a^					0.139
1	35 (5.5)	11 (5.0)	17 (6.8)	7 (4.2)	
2	173 (27.3)	64 (29.4)	76 (30.5)	33 (19.8)	
3	414 (65.3)	138 (63.3)	153 (61.4)	123 (73.6)	
4	12 (1.9)	5 (2.3)	3 (1.2)	4 (2.4)	
CVD^b^	149 (23.4)	48 (21.9)	58 (23.3)	43 (25.6)	0.697
Arrythmias	62 (9.8)	18 (8.2)	19 (7.6)	25 (14.9)	0.032
History of DVT or PE	20 (3.1)	4 (1.8)	8 (3.2)	8 (4.8)	0.248
Respiratory diagnosis	240 (37.7)	70 (32.0)	85 (34.1)	85 (50.6)	< 0.001
Diabetes mellitus	169 (26.6)	59 (26.9)	62 (24.9)	48 (28.6)	0.669
CVA or TIA	32 (5.0)	10 (4.6)	8 (3.2)	14 (8.3)	0.059
HLD, dyslipidemia, or hypertriglyceridemia	305 (48.0)	89 (40.6)	122 (49.0)	94 (56.0)	0.011
HTN	334 (52.5)	128 (58.4)	117 (47.0)	89 (53.0)	0.046
Most recent WBC, median [IQR], × 10^9^/L	7.5 [5.9, 9.8]	7.5 [5.9, 9.5]	7.5 [6.0, 10.2]	7.6 [5.8, 10.2]	0.634
Albumin, median [IQR] (n = 632), g/dL^a^	4.1 [3.6, 4.3]	4.0 [3.6, 4.2]	4.1 [3.6, 4.4]	4.1 [3.7, 4.4]	0.005
BMI, median [IQR] (n = 635), kg/m^2 a^	7.5 [5.9, 9.8]	7.5 [5.9, 9.5]	7.5 [6.0, 10.2]	7.6 [5.8, 10.2]	0.634
Surgical indication					0.001
Acute cholecystitis	211 (33.2)	51 (23.3)	99 (39.8)	61 (36.3)	
Chronic cholecystitis only	133 (20.9)	57 (26.0)	34 (13.6)	42 (25.0)	
Dropped stone only^c^	108 (17.0)	39 (17.8)	40 (16.1)	29 (17.3)	
Concern for gallbladder malignancy	4 (0.6)	2 (0.9)	1 (0.4)	1 (0.6)	
Other benign indication	168 (29.1)	70 (32.0)	75 (30.1)	23 (21.1)	

*IQR* interquartile range, *ASA* American Society of Anesthesiologists classification system, *CVD* cardiovascular disease, *DVT* deep vein thrombosis, *PE* pulmonary embolism, *CVA* cerebrovascular accident, *TIA* transient ischemic attack, *HLD* hyperlipidemia, *HTN* hypertension, *CKD* chronic kidney disease, *ESRD* end-stage renal disease, *HD* hemodialysis, *WBC* white blood cell count, *BMI* body mass index

^a^Not recorded in electronic medical record of all patients

^b^Includes heart failure, valvular and non-valvular disease, peripheral artery disease, and aneurysms

^c^Includes disease processes with stones outside of the gallbladder such as choledocholithiasis and/or gallstone pancreatitis

Prior to the introduction of the robot (pre-robotic era), 97.3% of surgeries were initiated laparoscopy with a conversion rate of 14.6%. During the transition period 53.0% of surgeries were initiated laparoscopically with a 7.6% conversion rate and 47.0% robotically with a 0.0% conversion rate. All surgeries during the robotic era were performed robotically with a 0.0% conversion rate (*p* <  0.001) and no difference in rates of subtotal cholecystectomies (1.8% in the robotic era vs 5.0% in the pre-robotic era and 2.4% in the transition era, *p* = 0.136). Compared to the pre-robotic era, more patients underwent non-elective surgery in the robotic era (*p* <  0.001). There was no difference in percutaneous cholecystostomy tubes removed intra-operatively, intra-operative complications, drain placement, and operative time between all three cohorts. On final pathology significantly more patients had acute cholecystitis in the robotic era. See Table 2 for operative characteristics between cohorts (complete operative characteristics in Supplemental Table 2).

**Table 2:** Operative characteristics

Characteristics	Total (%)	Pre-robotic era (%)	Transition era (%)	Robotic era (%)	*p* value
	636 (100.0)	219 (34.4)	249 (39.2)	168 (26.4)	
*Operative characteristics*					
Non-elective scheduled surgery	199 (31.3)	39 (17.8)	90 (36.1)	70 (41.7)	< 0.001
Surgical technique					< 0.001
Robotic	285 (44.8)	0 (0.0)	117 (47.0)	168 (100.0)	
Laparoscopic	304 (47.8)	182 (83.1)	122 (49.0)	0 (0.0)	
Primary open	6 (0.9)	6 (2.7)	0 (0.0)	0 (0.0)	
Laparoscopic-converted-to-open	41 (6.9)	31 (14.2)	10 (4.0)	0 (0.0)	
Conversion (n = 630)^a^	41 (6.5)	31 (14.6)	10 (4.0)	0 (0.0)	< 0.001
By novice robotic surgeon (n = 285 robotic cases)	177 (62.1)	-	62 (53.0)	115 (68.4)	0.008
Intra-operative cholangiogram	20 (3.1)	16 (7.3)	4 (1.6)	0 (0.0)	< 0.001
Secondary procedure performed^b^	90 (14.2)	29 (13.2)	35 (14.1)	26 (15.5)	0.821
Percutaneous cholecystostomy removed	39 (6.1)	10 (4.6)	15 (6.0)	14 (8.3)	0.333
Subtotal performed	20 (3.1)	11 (5.0)	6 (2.4)	3 (1.8)	0.136
Wound classification					0.021
Clean	0 (0.0)	0 (0.0)	0 (0.0)	0 (0.0)	
Clean/contaminated	272 (42.8)	107 (48.9)	93 (37.4)	72 (42.9)	
Contaminated	284 (44.6)	96 (43.8)	116 (46.6)	72 (42.9)	
Dirty	80 (12.6)	16 (7.3)	40 (16.1)	24 (14.3)	
Pre-operative antibiotics given	182 (28.6)	47 (21.5)	81 (32.5)	54 (32.1)	0.015
Operative time, median [IQR] (n = 634), min^cd^	120 [94, 162]	122 [89, 167]	118 [93, 165]	123 [100, 152]	0.911
Estimated blood loss, median [IQR], mL	10 [15, 25]	20 [10, 50]	10 [5, 25]	10 [5, 15]	< 0.001
Final pathology					0.003
Acute cholecystitis	234 (36.8)	58 (26.5)	101 (40.6)	75 (44.6)	
Chronic cholecystitis only	391 (61.5)	156 (71.2)	145 (58.2)	90 (53.6)	
All other benign	11 (1.7)	5 (2.3)	3 (1.2)	3 (1.8)	

*IQR* interquartile range

^a^Conversion to open cholecystectomy excluding 6 open cholecystectomies

^b^Excludes primary open umbilical hernia repairs through trocar site and minor procedures

^c^Operative time refers to the time the patient entered the operating room to the time the patient exited the operating room

^d^Not recorded in electronic medical record of all patients

There were no bile duct injuries in any cohort during the study period. There was no difference in complications (includes superficial or deep surgical site infections, biliary leak, and/or retained stones) between cohorts. Post-operative procedures, and 30-day emergency department (ED) visits and re-admissions were similar. Fewer blood transfusions (post-operative day 0 to 30) were given in the robotic era. Pre- and post-operative length of stay was statistically significantly different between the three cohorts (*p* = 0.003 and *p* = 0.013, respectively). See Table 3 for post-operative outcomes between cohorts (complete post-operative characteristics in Supplemental Table 3).

**Table 3:** Post-operative outcomes

Characteristics	Total	Pre-robotic era (%)	Transition era (%)	Robotic era (%)	*p* value
	636 (100.0)	219 (34.4)	249 (39.2)	168 (26.4)	
*Post-operative outcomes*					
Received transfusion (POD 0–30 days)	17 (2.7)	8 (3.6)	9 (3.6)	0 (0.0)	0.043
Post-operative antibiotics given	137 (21.5)	54 (24.7)	54 (21.7)	29 (17.3)	0.214
Complication (POD 0–30 days)^a^	41 (6.4)	21 (9.6)	12 (4.8)	8 (4.8)	0.065
Length of stay, mean (SD)					
Pre-operative	1.0 (2.4)	0.9 (3.0)	1.2 (2.4)	0.7 (1.4)	0.003
Post-operative	2.2 (3.2)	2.2 (2.5)	2.4 (3.5)	1.8 (3.3)	0.013
Re-admission(s) 30 days after discharge	32 (5.0)	14 (6.4)	12 (4.8)	6 (3.6)	0.444
ED visit(s) 30 days after discharge	105 (16.5)	36 (16.4)	44 (17.7)	25 (14.9)	0.753
Additional procedure(s) (POD 0–30 days)	27 (4.2)	14 (6.4)	8 (3.2)	5 (3.0)	0.149
ERCP/EUS performed (POD 0–30 days)	19 (3.0)	9 (4.1)	6 (2.4)	4 (2.4)	0.490
IR procedures performed (POD 0–30 days)	13 (2.2)	7 (3.2)	5 (2.4)	1 (0.6)	0.202
Re-operations (POD 0–30 days)	4 (0.6)	1 (0.5)	2 (0.8)	1 (0.6)	1.000
Number of post-operative visits					0.118
0	97 (15.2)	39 (17.8)	29 (11.6)	29 (17.3)	
1	461 (72.5)	147 (67.1)	192 (77.1)	122 (72.6)	
≥ 2	78 (12.3)	33 (15.1)	28 (11.2)	17 (10.1)	
Discharged from clinic after first visit	463 (72.9)	151 (69.0)	191 (76.7)	121 (72.5)	0.167

*IQR* interquartile range, *SSI* surgical site infection, *SD* standard deviation, *ED* emergency department, *POD* post-operative day, *ERCP* endoscopic retrograde cholangiopancreatography, *EUS* endoscopic ultrasound, *IR* interventional radiology

^a^Complications include: superficial and deep surgical site infections, biliary leak, and/or retained stone of sludge

On multivariable analysis, the transition and robotic eras compared to the pre-robotic era did not affect the odds of complication (superficial and deep surgical site infections, retained stone or sludge, and/or biliary leak), 30-day re-admission, or 30-day emergency department visit (see Table 4). Only conversion to open surgery was associated with an increased odds of the complications mentioned above (aOR 3.45 95% CI [1.24, 9.56]). See Supplemental Fig. 1a–c for complete multivariable analysis.

**Table 4:** Multivariable analysis of post-operative outcomes

Adjusted outcome	By surgical eras			Odds ratio [95% CI] (baseline pre-robotic era)	
	Pre-robotic era (%)	Transition era (%)	Robotic era (%)	Transition era (%)	Robotic era (%)
Complication^a^	9.6	4.8	4.8	0.38 [0.13, 1.12]	0.31 [0.07, 1.42]
Re-admission^b^	6.4	4.8	3.6	0.38 [0.10, 1.41]	0.23 [0.04, 1.38]
ED presentation^b^	16.4	17.7	14.9	0.81 [0.42, 1.57]	0.57 [0.22, 1.45]

^a^Complications included: superficial and deep surgical site infections, biliary leak, and/or retained stone of sludge. Outcomes reflect age, sex, diabetes, ASA classification, wound classification, surgical indication, surgical eras, surgical approach, percutaneous cholecystostomy tube, conversion to open; n = 625

^b^Outcomes reflect age, sex, diabetes, ASA classification, wound classification, surgical indication, surgical approach, surgical eras, percutaneous cholecystostomy tube, conversion to open, post-operative complication, post-operative length of stay; re-admission analysis n = 627; ED presentation analysis n = 589

### Robotic compared to composite of non-robotic cholecystectomy techniques

On univariate analysis robotic cholecystectomy had a conversion rate of 0.0% vs 11.9% in the non-robotic cohort (*p* <  0.001). There were no differences in subtotal cholecystectomy rates and post-operative outcomes, including 30-day re-admissions and 30-day emergency department presentations. See Supplemental Tables 4–6.

Subgroup analysis was performed on patients with the highest risk of conversion (i.e., patients with acute cholecystitis and with percutaneous cholecystostomy tubes). In patients with acute cholecystitis (n = 211) there were no conversions in the robotic cohort compared to 25 (23.8%, *p* <  0.001) of cases started laparoscopically with a number needed to treat of 4.2. Operative time was less in the robotic cohort (median time 125 min vs 157.5 min, *p* = 0.010). Fewer patients received post-operative antibiotics in the robotic cohort (29.8% vs 52.3%, *p* = 0.001). Mean post-operative length of stay was 1.7 days shorter (2.4 days ± 3.2 vs 4.1 days ± 4.8; *p* = 0.001) (see Supplemental 7–9). On multivariable analysis, conversion to open cholecystectomy was associated with higher odds of post-operative complication (aOR 4.74 95% CI [1.15, 19.47]) (see Supplemental Fig. 2a–c and Supplemental Table 13).

On subgroup analysis of patients with percutaneous cholecystostomy tubes (n = 39) at the time of surgery, the conversion rate was 0.0% for robotic cholecystectomy vs 21.4% for laparoscopic cholecystectomy (*p* = 0.043). Operative time was less in the robotic cohort (median time 130 min vs 229 min, *p* <  0.001; see Supplemental Tables 10–12). Fewer patients received post-operative antibiotics (*p* = 0.031) and were re-admitted within 30 days post-operatively (0.0% vs 26.7%, *p* = 0.017). The mean post-operative length of stay was 1.7 days shorter (1.8 days ± 1.4 vs 3.5 days ± 2.5; *p* = 0.014) (see Supplemental Fig. 3a-b and Supplemental Table 13).

## Discussion

From 2016 to 2021, the GLA VA hospital experienced a paradigm shift in the surgical management of benign gallbladder disease such that by the end of 2021 all cholecystectomies were performed robotically regardless of pre-operative and patient factors. As the robotic platform increased in use, the rates of conversion and primary open surgery decreased. Strikingly, there were zero conversions to open in the robotic cohort with equivalent rates of subtotal cholecystectomies and peri-operative complications (including no bile duct injuries) compared to the other two eras; rates of surgical site infections, biliary leaks, retained stones or sludge, and 30-day re-admissions and emergency department presentations were similar between cohorts. Notably, peri-operative outcomes were superior in the robotic cohort for the subgroups of patients with acute cholecystitis and percutaneous cholecystostomy tubes.

Though conversion to open cholecystectomy is not a surgical complication, rather an approach to reduce intra-operative injury and ensure patient safety, it is associated with higher odds of complications [6]. Randomized controlled trials demonstrated increased morbidity in patients with acute cholecystitis undergoing open cholecystectomy compared to minimally invasive surgery [3]. Therefore, it behooves surgeons and patients alike to avoid open cholecystectomy if possible. Factors known to increase the risk of conversion are well known and include age over 40 years, acute cholecystitis, male sex, and percutaneous cholecystostomy tubes (conversion rates of 8–41%) [9, 14]. Conversion rates during robotic cholecystectomy have been reported between 0 and 3.9% [9, 18–21] and were either equivalent or superior to conversion rates during laparoscopic surgery for benign gallbladder disease on meta-analysis [17, 22]. While there are other studies that report 0% conversion rates with robotic cholecystectomy [20, 21], their generalizability is often limited by patient selection. Of those reporting outcomes of consecutive robotic cholecystectomies, more advanced benign gallbladder disease such as acute and gangrenous or perforated cholecystitis is often excluded [23, 24]. In addition, these studies frequently included lower risk populations and were reported as a single surgeon experience [25, 26]. This is in contrast to our study in which we included all patients regardless of disease severity and who underwent surgery by multiple surgeons.

Within our robotic cohort, the previously mentioned risk factors for conversion were significantly more common than reported in previous studies that evaluate robotic cholecystectomy conversions in the veteran population; 87.4% versus 74% of patients were male, 36.5% versus 8% had a pre-operative diagnosis of acute cholecystitis, and 8.4% (not reported in other studies) of patients had a percutaneous cholecystostomy tube in place [8]. Even in our particularly high-risk subgroup analyses (acute cholecystitis and percutaneous cholecystostomy subgroups), there were no conversions from robotic to open cholecystectomy without an increase in subtotal cholecystectomies being performed. Our data more clearly establishes the superior role of the robotic platform in the care of patients with clinicopathologic features defining a high-risk population for conversion.

The findings in this study are reported amid skepticism within the surgical community of transitioning to the robotic platform for cholecystectomies. Katala, et al. [27] compared robotic to laparoscopic cholecystectomies performed between 2010 and 2019 reporting significantly higher rates of bile duct injury with a relative risk of 3.16. However, bile duct injury rates were not compared in the early and later years of robotic use, a period over which there was a 37-fold increase in robotic cholecystectomies. This is an important caveat as surgeons only began utilizing the robotic platform for cholecystectomies around 2010 [28, 29]. Numerous studies show increased surgical volume is associated with decreased complications and improved outcomes [13, 30, 31]. In particular, a recent *JAMA Surgery* publication reported “[b]ile duct injury rates following robotic-assisted cholecystectomy decreased with increasing experience” [32]. Importantly, our current study found no such issue with learning curve or volume of procedures during the 13 year adoption of the robotic platform demonstrating that robotic cholecystectomy can be introduced without increased morbidity. In particular, the highest risk patients for conversion and complication benefited the most from the utilization of the robotic platform. Thus, the findings herein provide the clinical justification to devote time and resources to developing a robotic program for management of these patients.

While cost is often cited as a barrier to adopting the robotic platform—approximately $2.6 million USD for the da Vinci Xi—the reduction in open and conversion-to-open cholecystectomies offers a compelling financial offset [33]. Each conversion to open adds an estimated $8,500 USD compared to even the longest laparoscopic cases [34]. Given the historically high conversion rate in the VA population (14.6% prior to robotic adoption), the robotic platform presents an opportunity to significantly reduce both conversion-related costs and the need for planned open procedures, ultimately decreasing overall hospital expenditures. As such, our study fills two knowledge gaps. First, we report the safe adoption of the robotic platform for elective and acute care management of benign gallbladder disease in a VA population. Second, we show the specific benefit of the robotic approach to the management of complex and high-risk gallbladder disease.

The strength of our study is that it reflects the practical adoption of the robotic platform to perform cholecystectomies for benign gallbladder disease, from selective initiation to complete, non-selective use. Our early favorable results have fueled the continued adoption of the robotic platform such that all surgeons were trained to perform robotic cholecystectomies, and all surgeons now prefer the robotic platform uniformly for all cases. Moreover, patients were neither selected according to patient and pathologic factors nor the surgery start time. In other words, cholecystectomies were performed robotically during daytime hours and overnight according to the clinical necessity. In large part this was due to the VA hospital system accommodating access to the robotic platform for acute care surgery for gallbladder disease.

In addition, our data reflect cholecystectomies performed by multiple surgeons, most of whom were novice robotic surgeons at the start of the transition to the robotic platform, bolstering our findings of a 0% conversion rate. While the GLA VA hospital does have physician assistants trained in robotic assistance, they do not partake in general surgery cases. Significant buy-in from general surgeons and operative staff in addition to surgeon familiarity and comfort with robotic surgery and robot setup and docking made this paradigm shift feasible. Importantly, the work required for implementing this paradigm shift should not be understated. Training nursing personnel and securing buy-in from operative staff and anesthesia teams was a critical and time-intensive process, requiring approximately five months of ongoing dialogue and protocol development. This may also explain the comparable operative times and even the shorter operative times within the acute cholecystitis and percutaneous cholecystostomy cohorts. Lastly, our low conversion rate may be attributed to the collaborative practice of the GLA VA surgeons who often make themselves available for challenging cases, a practice ongoing throughout the entirety of the study period. Future studies evaluating the learning curve as well as outcomes of robotic cholecystectomies performed by individual GLA VA surgeons, each of which has a varying degree of robotic experience, would be beneficial to further understand the outcome-experience relationship.

This study has several limitations. First, this retrospective chart review relied upon accurate documentation within operative notes and patient notes, thus was subject to data omission and inaccuracies. Second, though the VA electronic medical record (CPRS) often contained documentation when patients received healthcare at an outside facility, this was not necessarily always reported, and patients may have presented to other facilities with complications that were not captured in our review.

This study evaluates a paradigm shift in the treatment of benign gallbladder disease with the goal of eliminating the morbidity associated with open cholecystectomies. We compared the outcomes and safety of cholecystectomies before, during, and after a paradigm shift in management towards the robotic approach. We report the elimination of conversions to open cholecystectomy during and after the paradigm shift and by using a robotic technique without an increase in the use of “bailout” techniques such as subtotal cholecystectomies, and without any negative impact on operative or post-operative outcomes. This is one of the largest studies evaluating the adoption of robotic cholecystectomy in a high-risk, predominantly male, VA population. Given our data, we were able to prove our hypothesis of maintaining optimal surgical quality while eliminating open cholecystectomy using the robotic approach. Therefore, we recommend the adoption of the robotic platform for the patients at highest risk for conversion and those with acute cholecystitis and percutaneous cholecystostomy tubes.

## Supplementary Information

Below is the link to the electronic supplementary material.

Supplementary file1 (DOCX 932 KB)

## References

[1] Barkun JS, Barkun AN, Sampalis JS et al (1992) Randomised controlled trial of laparoscopic versus mini cholecystectomy. The McGill Gallstone Treatment Group. Lancet 340(8828):1116–1119. 10.1016/0140-6736(92)93148-g 1359210 10.1016/0140-6736(92)93148-g

[2] Hendolin HI, Paakonen ME, Alhava EM, Tarvainen R, Kemppinen T, Lahtinen P (2000) Laparoscopic or open cholecystectomy: a prospective randomised trial to compare postoperative pain, pulmonary function, and stress response. Eur J Surg 166(5):394–399. 10.1080/110241500750008961 10881952 10.1080/110241500750008961

[3] Kiviluoto T, Siren J, Luukkonen P, Kivilaakso E (1998) Randomised trial of laparoscopic versus open cholecystectomy for acute and gangrenous cholecystitis. Lancet 351(9099):321–325. 10.1016/S0140-6736(97)08447-X 9652612 10.1016/S0140-6736(97)08447-X

[4] Coccolini F, Catena F, Pisano M et al (2015) Open versus laparoscopic cholecystectomy in acute cholecystitis. Systematic review and meta-analysis. Int J Surg 18:196–204. 10.1016/j.ijsu.2015.04.083 25958296 10.1016/j.ijsu.2015.04.083

[5] Antoniou SA, Antoniou GA, Koch OO, Pointner R, Granderath FA (2014) Meta-analysis of laparoscopic vs open cholecystectomy in elderly patients. World J Gastroenterol 20(46):17626–17634. 10.3748/wjg.v20.i46.17626 25516678 10.3748/wjg.v20.i46.17626 PMC4265625

[6] Kaafarani HMA, Smith TS, Neumayer L, Berger DH, DePalma RG, Itani KMF (2010) Trends, outcomes, and predictors of open and conversion to open cholecystectomy in Veterans Health Administration hospitals. Am J Surg 200(1):32–40. 10.1016/j.amjsurg.2009.08.020 20637334 10.1016/j.amjsurg.2009.08.020

[7] Sabour AF, Matsushima K, Love BE et al (2020) Nationwide trends in the use of subtotal cholecystectomy for acute cholecystitis. Surgery 167(3):569–574. 10.1016/j.surg.2019.11.004 31879089 10.1016/j.surg.2019.11.004

[8] Tao Z, Emuakhagbon VS, Pham T, Augustine MM, Guzzetta A, Huerta S (2021) Outcomes of robotic and laparoscopic cholecystectomy for benign gallbladder disease in Veteran patients. J Robot Surg 15(6):849–857. 10.1007/s11701-020-01183-3 33400103 10.1007/s11701-020-01183-3

[9] Gangemi A, Danilkowicz R, Bianco F, Masrur M, Giulianotti PC (2017) Risk factors for open conversion in minimally invasive cholecystectomy. JSLS. 10.4293/JSLS.2017.00062 29238153 10.4293/JSLS.2017.00062 PMC5714218

[10] Albisher HM, Foula MS, Alghusnah ES, Abdelhafiz T (2023) Risk factors and outcomes in acute perforated gallbladder: a retrospective cohort study. Asian J Surg 46(6):2299–2303. 10.1016/j.asjsur.2022.09.109 36229304 10.1016/j.asjsur.2022.09.109

[11] Livingston EH, Rege RV (2004) A nationwide study of conversion from laparoscopic to open cholecystectomy. Am J Surg 188(3):205–211. 10.1016/j.amjsurg.2004.06.013 15450821 10.1016/j.amjsurg.2004.06.013

[12] Oymaci E, Ucar AD, Aydogan S, Sari E, Erkan N, Yildirim M (2014) Evaluation of affecting factors for conversion to open cholecystectomy in acute cholecystitis. Prz Gastroenterol 9(6):336–341. 10.5114/pg.2014.45491 25653728 10.5114/pg.2014.45491 PMC4300343

[13] Huy TC, Shenoy R, Russell MM, Girgis M, Tomlinson JS (2024) Patient and hospital factors influence surgical approach in treatment of acute cholecystitis. Surg Endosc. 10.1007/s00464-024-11227-6 39285035 10.1007/s00464-024-11227-6

[14] Bickel A, Hoffman RS, Loberant N, Weiss M, Eitan A (2016) Timing of percutaneous cholecystostomy affects conversion rate of delayed laparoscopic cholecystectomy for severe acute cholecystitis. Surg Endosc 30(3):1028–1033. 10.1007/s00464-015-4290-y 26139479 10.1007/s00464-015-4290-y

[15] Kourounis G, Rooke ZC, McGuigan M, Georgiades F (2022) Systematic review and meta-analysis of early vs late interval laparoscopic cholecystectomy following percutaneous cholecystostomy. HPB (Oxford) 24(9):1405–1415. 10.1016/j.hpb.2022.03.016 35469743 10.1016/j.hpb.2022.03.016

[16] Kong J, Shahait A, Girten K et al (2021) Recent trends in cholecystectomy in US veterans. Surg Endosc 35(10):5558–5566. 10.1007/s00464-020-08056-8 33025254 10.1007/s00464-020-08056-8

[17] Huang Y, Chua TC, Maddern GJ, Samra JS (2017) Robotic cholecystectomy versus conventional laparoscopic cholecystectomy: a meta-analysis. Surgery 161(3):628–636. 10.1016/j.surg.2016.08.061 28011011 10.1016/j.surg.2016.08.061

[18] Reinisch A, Liese J, Padberg W, Ulrich F (2023) Robotic operations in urgent general surgery: a systematic review. J Robot Surg 17(2):275–290. 10.1007/s11701-022-01425-6 35727485 10.1007/s11701-022-01425-6 PMC10076409

[19] Ayloo S, Roh Y, Choudhury N (2014) Laparoscopic versus robot-assisted cholecystectomy: a retrospective cohort study. Int J Surg 12(10):1077–1081. 10.1016/j.ijsu.2014.08.405 25218366 10.1016/j.ijsu.2014.08.405

[20] Cho G, Yoo T, Chang W (2022) Robotic cholecystectomy with a new port placement: is it really beneficial? Asian J Surg 45(8):1542–1546. 10.1016/j.asjsur.2021.09.016 34742622 10.1016/j.asjsur.2021.09.016

[21] Main WPL, Mitko JM, Hussain LR, Meister KM, Kerlakian GM (2017) Robotic versus Laparoscopic Cholecystectomy in the Obese Patient. Am Surg 83(11):e447–e449 30454233

[22] Straatman J, Pucher PH, Knight BC et al (2023) Systematic review: robot-assisted versus conventional laparoscopic multiport cholecystectomy. J Robot Surg 17(5):1967–1977. 10.1007/s11701-023-01662-3 37439902 10.1007/s11701-023-01662-3

[23] Chowbey P, Dewan A, Sharma A, Khullar R, Soni V, Baijal M (2023) A review of the first 100 robotic cholecystectomies with a new cart-based surgical robot at a tertiary care centre. J Minim Access Surg 19(3):390–394. 10.4103/jmas.jmas_184_22 37282423 10.4103/jmas.jmas_184_22 PMC10449052

[24] Breitenstein S, Nocito A, Puhan M, Held U, Weber M, Clavien PA (2008) Robotic-assisted versus laparoscopic cholecystectomy: outcome and cost analyses of a case-matched control study. Ann Surg 247(6):987–993. 10.1097/SLA.0b013e318172501f 18520226 10.1097/SLA.0b013e318172501f

[25] Khanna S, Barua A (2022) Robotic assisted cholecystectomy—a retrospective cohort study of experience of 106 first robotic cholecystectomies in versius robotic platform. Int J Surg Open 47:100554. 10.1016/j.ijso.2022.100554

[26] Corvo PR, Bendl RF (2014) One hundred consecutive robotically assisted cholecystectomies by one surgeon without any conversions to an open technique. J Robot Surg 8(3):251–254. 10.1007/s11701-014-0461-4 27637686 10.1007/s11701-014-0461-4

[27] Kalata S, Thumma JR, Norton EC, Dimick JB, Sheetz KH (2023) Comparative safety of robotic-assisted vs laparoscopic cholecystectomy. JAMA Surg 158(12):1303–1310. 10.1001/jamasurg.2023.4389 37728932 10.1001/jamasurg.2023.4389 PMC10512167

[28] Aguayo E, Dobaria V, Nakhla M et al (2020) National trends and outcomes of inpatient robotic-assisted versus laparoscopic cholecystectomy. Surgery 168(4):625–630. 10.1016/j.surg.2020.06.018 32762874 10.1016/j.surg.2020.06.018

[29] Sheetz KH, Claflin J, Dimick JB (2020) Trends in the adoption of robotic surgery for common surgical procedures. JAMA Netw Open 3(1):e1918911. 10.1001/jamanetworkopen.2019.18911 31922557 10.1001/jamanetworkopen.2019.18911 PMC6991252

[30] Anderson JE, Chang DC (2014) Does the effect of surgical volume on outcomes diminish over time? JAMA Surg 149(4):398–400. 10.1001/jamasurg.2013.4654 24500686 10.1001/jamasurg.2013.4654

[31] Blohm M, Sandblom G, Enochsson L, Hedberg M, Andersson MF, Osterberg J (2023) Relationship between surgical volume and outcomes in elective and acute cholecystectomy: nationwide, observational study. Br J Surg 110(3):353–361. 10.1093/bjs/znac415 36422988 10.1093/bjs/znac415 PMC10364541

[32] Sheetz KH, Thumma JR, Kalata S, Norton EC, Dimick JB (2024) Learning curve for robotic-assisted cholecystectomy. JAMA Surg 159(7):833–836. 10.1001/jamasurg.2024.1221 38776095 10.1001/jamasurg.2024.1221 PMC11112490

[33] Ho C, Tsakonas E, Tran K et al (2011) Robot-assisted surgery compared with open surgery and laparoscopic surgery: clinical effectiveness and economic analyses. CADTH Health Technology Assessments 24175355

[34] Lengyel BI, Panizales MT, Steinberg J, Ashley SW, Tavakkoli A (2012) Laparoscopic cholecystectomy: what is the price of conversion? Surgery 152(2):173–178. 10.1016/j.surg.2012.02.016 22503324 10.1016/j.surg.2012.02.016 PMC3667156

