# Supplementary material for: The robotic era: 11-year retrospective study of cholecystectomies at a veterans affairs hospital
Source: Surg Endosc. 2025 Sep 4;39(11):7476–85. doi: 10.1007/s00464-025-12185-3 (PMC12618365; doi:10.1007/s00464-025-12185-3)
Supplement: Supplementary file 1 — Supplementary file1 (DOCX 932 KB) [file 464_2025_12185_MOESM1_ESM.docx]

Table 1. Demographic and clinical characteristics between surgical eras.

| **Characteristics** | **Total (%)** | **Pre-robotic era (%)** | **Transition era (%)** | **Robotic era (%)** | **p-value** |
| --- | --- | --- | --- | --- | --- |
|  | 636 (100.0) | 219 (34.4) | 249 (39.2) | 168 (26.4) |  |
| **Patient Characteristics** | | | | | |
| Age, median [IQR] | 61 [46, 70] | 60 [46, 67] | 62 [44, 70] | 61 [47, 74] | 0.195 |
| Sex, male | 547 (86.0) | 194 (88.6) | 205 (82.3) | 148 (88.1) | 0.099 |
| Race |  |  |  |  | 0.503 |
| *White* | 412 (64.8) | 147 (67.1) | 155 (62.2) | 110 (65.5) |  |
| *Black or African American* | 115 (18.1) | 43 (19.6) | 45 (18.1) | 27 (16.1) |  |
| *Asian* | 249 (4.6) | 7 (3.2) | 11 (4.4) | 11 (6.6) |  |
| *American Indian or Alaskan Native* | 3 (0.5) | 2 (0.9) | 0 (0.0) | 1 (0.6) |  |
| *Native Hawaiian or other Pacific Islander* | 9 (1.4) | 2 (0.9) | 4 (1.6) | 3 (1.8) |  |
| *Two or more races* | 4 (0.6) | 1 (0.5) | 3 (1.2) | 0 (0.0) |  |
| *Declined or unknown or unanswered* | 64 (10.1) | 17 (7.8) | 31 (12.4) | 16 (9.5) |  |
| Ethnicity |  |  |  |  | 0.527 |
| *Not Hispanic of Latino* | 420 (66.0) | 147 (67.1) | 158 (63.4) | 115 (68.4) |  |
| *Hispanic of Latino* | 177 (27.8) | 60 (27.4) | 71 (28.5) | 46 (27.4) |  |
| *Declined or unknown* | 39 (6.1) | 12 (5.5) | 20 (8.0) | 7 (4.2) |  |
| History of prior surgery |  |  |  |  | 0.091 |
| *Abdominal* | 204 (32.1) | 75 (34.2) | 66 (26.5) | 63 (37.5) |  |
| *Non-abdominal or none* | 427 (67.1) | 143 (65.3) | 181 (72.7) | 103 (61.3) |  |
| *Unknown* | 5 (0.8) | 1 (0.5) | 2 (0.8) | 2 (1.2) |  |
| Tobacco use^a^ | 126 (19.8) | 54 (24.7) | 46 (18.5) | 26 (15.5) | 0.064 |
| Marijuana use^a^ | 63 (9.9) | 22 (10.0) | 21 (8.4) | 20 (11.9) | 0.506 |
| Alcohol use disorder | 32 (5.6) | 14 (6.4) | 13 (5.2) | 5 (4.6) | 0.763 |
| ASA (n=634)^a^ |  |  |  |  | 0.139 |
| *1* | 35 (5.5) | 11 (5.0) | 17 (6.8) | 7 (4.2) |  |
| *2* | 173 (27.3) | 64 (29.4) | 76 (30.5) | 33 (19.8) |  |
| *3* | 414 (65.3) | 138 (63.3) | 153 (61.4) | 123 (73.6) |  |
| *4* | 12 (1.9) | 5 (2.3) | 3 (1.2) | 4 (2.4) |  |
| CVD^b^ | 149 (23.4) | 48 (21.9) | 58 (23.3) | 43 (25.6) | 0.697 |
| Arrythmias | 62 (9.8) | 18 (8.2) | 19 (7.6) | 25 (14.9) | 0.032 |
| History of DVT or PE | 20 (3.1) | 4 (1.8) | 8 (3.2) | 8 (4.8) | 0.248 |
| Respiratory Diagnosis | 240 (37.7) | 70 (32.0) | 85 (34.1) | 85 (50.6) | <0.001 |
| Diabetes mellitus | 169 (26.6) | 59 (26.9) | 62 (24.9) | 48 (28.6) | 0.669 |
| CVA or TIA | 32 (5.0) | 10 (4.6) | 8 (3.2) | 14 (8.3) | 0.059 |
| HLD, dyslipidemia, or hypertriglyceridemia | 305 (48.0) | 89 (40.6) | 122 (49.0) | 94 (56.0) | 0.011 |
| HTN | 334 (52.5) | 128 (58.4) | 117 (47.0) | 89 (53.0) | 0.046 |
| Cirrhosis | 21 (3.3) | 7 (3.2) | 7 (2.8) | 7 (4.2) | 0.745 |
| Liver disease excluding cirrhosis | 148 (23.3) | 44 (20.1) | 65 (26.1) | 39 (23.2) | 0.307 |
| Renal disease |  |  |  |  | 0.240 |
| *None* | 546 (85.8) | 195 (89.0) | 207 (83.1) | 144 (85.7) |  |
| *CKD* | 85 (13.4) | 22 (10.0) | 41 (16.5) | 22 (13.1) |  |
| *ESRD on HD* | 5 (0.8) | 2 (0.9) | 1 (0.4) | 2 (1.2) |  |
| WBC at presentation, median [IQR] (n=228), x10^9^/L^e^ | 11.3 [8.3, 14.4] | 11.0 [8.6, 15.3] | 11.3 [8.0, 14.1] | 11.5 [8.6, 14.0] | 0.928 |
| Most recent WBC, median [IQR], x10^9^/L | 7.5 [5.9, 9.8] | 7.5 [5.9, 9.5] | 7.5 [6.0, 10.2] | 7.6 [5.8, 10.2] | 0.634 |
| A1c (all), median [IQR] (n=596), %^b^ | 5.7 [5.3, 6.3] | 5.8 [5.4, 6.2] | 5.7 [5.4, 6.3] | 5.6 [5.3, 6.3] | 0.768 |
| A1c <3 mths pre-op, median [IQR] (n=421), %^b^ | 5.7 [5.4, 6.4] | 5.7 [5.4, 6.2] | 5.7 [5.4, 6.4] | 5.6 [5.3, 6.5] | 0.949 |
| Albumin, median [IQR] (n=632), g/dL^b^ | 4.1 [3.6, 4.3] | 4.0 [3.6, 4.2] | 4.1 [3.6, 4.4] | 4.1 [3.7, 4.4] | 0.005 |
| Total bilirubin, median [IQR] (n=635), mg/dL^b^ | 0.8 [0.5, 1.1] | 0.8 [0.6, 1.1] | 0.8 [0.5, 1.1] | 0.7 [0.5, 1.2] | 0.662 |
| BMI, median [IQR] (n=635), kg/m^2 b^ | 7.5 [5.9, 9.8] | 7.5 [5.9, 9.5] | 7.5 [6.0, 10.2] | 7.6 [5.8, 10.2] | 0.634 |

| **Characteristics (continued)** | **Total (%)** | **Pre-robotic era (%)** | **Transition era (%)** | **Robotic era (%)** | **p-value** |
| --- | --- | --- | --- | --- | --- |
|  | 636 (100.0) | 219 (34.4) | 249 (39.2) | 168 (26.4) |  |
| **Patient Characteristics** | | | | | |
| Surgical Indication |  |  |  |  | 0.001 |
| *Acute cholecystitis* | 211 (33.2) | 51 (23.3) | 99 (39.8) | 61 (36.3) |  |
| *Chronic cholecystitis only* | 133 (20.9) | 57 (26.0) | 34 (13.6) | 42 (25.0) |  |
| *Dropped stone only*^c^ | 108 (17.0) | 39 (17.8) | 40 (16.1) | 29 (17.3) |  |
| *Concern for gallbladder malignancy* | 4 (0.6) | 2 (0.9) | 1 (0.4) | 1 (0.6) |  |
| *Other benign indication* | 168 (29.1) | 70 (32.0) | 75 (30.1) | 23 (21.1) |  |

Abbreviations: IQR interquartile range, ASA American Society of Anesthesiologists classification system, CVD cardiovascular disease, DVT deep vein thrombosis, PE pulmonary embolism, CVA cerebrovascular accident, TIA transient ischemic attack, HLD hyperlipidemia, HTN hypertension, CKD chronic kidney disease, ESRD end-stage renal disease, HD hemodialysis, WBC white blood cell count, BMI body mass index

^a^ Use within 8 weeks of surgery

^b^ Not recorded in electronic medical record of all patients.

^c^ Includes heart failure, valvular and non-valvular disease, peripheral artery disease, aneurysms.

^d^ Includes disease processes with stones outside of the gallbladder such as choledocholithiasis, gallstone pancreatitis.

^e^ Only obtained if patient admitted pre-operatively (excludes outpatients).

Table 2. Operative characteristics between surgery eras.

| **Characteristics** | **Total (%)** | **Pre-robotic era (%)** | **Transition era (%)** | **Robotic era (%)** | **p-value** |
| --- | --- | --- | --- | --- | --- |
|  | 636 (100.0) | 219 (34.4) | 249 (39.2) | 168 (26.4) |  |
| **Operative Characteristics** | | | | | |
| Surgery Schedule Type |  |  |  |  | <0.001 |
| *Elective* | 437 (68.7) | 180 (82.2) | 159 (63.9) | 98 (58.3) |  |
| *Non-elective* | 199 (31.3) | 39 (17.8) | 90 (36.1) | 70 (41.7) |  |
| Surgical Technique |  |  |  |  | <0.001 |
| *Robotic-assisted* | 285 (44.8) | 0 (0.0) | 117 (47.0) | 168 (100.0) |  |
| *Laparoscopic* | 304 (47.8) | 182 (83.1) | 122 (49.0) | 0 (0.0) |  |
| *Primary Open* | 6 (0.9) | 6 (2.7) | 0 (0.0) | 0 (0.0) |  |
| *Laparoscopic-converted-to-open* | 41 (6.9) | 31 (14.2) | 10 (4.0) | 0 (0.0) |  |
| Conversion (n=630)^a^ | 41 (6.5) | 31 (14.6) | 10 (4.0) | 0 (0.0) | <0.001 |
| By novice robotic surgeon (n=285 robotic cases) | 177 (62.1) | - | 62 (53.0) | 115 (68.4) | 0.008 |
| Intra-operative cholangiogram | 20 (3.1) | 16 (7.3) | 4 (1.6) | 0 (0.0) | <0.001 |
| Secondary procedure performed^b^ | 90 (14.2) | 29 (13.2) | 35 (14.1) | 26 (15.5) | 0.821 |
| *Percutaneous cholecystostomy removed* | 39 (6.1) | 10 (4.6) | 15 (6.0) | 14 (8.3) | 0.333 |
| Subtotal performed |  |  |  |  | 0.320 |
| *None* | 616 (96.9) | 208 (95.0) | 243 (97.6) | 165 (98.2) |  |
| *Fenestrating* | 4 (0.6) | 2 (0.9) | 2 (0.8) | 0 (0.0) |  |
| *Reconstituting* | 16 (2.5) | 9 (4.1) | 4 (1.6) | 3 (1.8) |  |
| Intra-operative complication | 5 (0.8) | 1 (0.5) | 3 (1.2) | 1 (0.6) | 0.744 |
| Wound classification |  |  |  |  | 0.021 |
| *Clean* | 0 (0.0) | 0 (0.0) | 0 (0.0) | 0 (0.0) |  |
| *Clean/contaminated* | 272 (42.8) | 107 (48.9) | 93 (37.4) | 72 (42.9) |  |
| *Contaminated* | 284 (44.6) | 96 (43.8) | 116 (46.6) | 72 (42.9) |  |
| *Dirty* | 80 (12.6) | 16 (7.3) | 40 (16.1) | 24 (14.3) |  |
| Pre-operative antibiotics given | 182 (28.6) | 47 (21.5) | 81 (32.5) | 54 (32.1) | 0.015 |
| Intra-operative drain placement, median [IQR] | 0 [0, 0] | 0 [0, 0] | 0 [0, 0] | 0 [0, 0] | 0.652 |
| Port placement, median [IQR] (n=625)^c^ | 4 [4, 4] | 4 [4, 4] | 4 [4, 4] | 4 [4, 5] | <0.001 |
| Operative time, median [IQR] (n=634), min^cd^ | 120 [94, 162] | 122 [89, 167] | 118 [93, 165] | 123 [100, 152] | 0.911 |
| Estimated blood loss, median [IQR], mL | 10 [15, 25] | 20 [10, 50] | 10 [5, 25] | 10 [5, 15] | <0.001 |
| Urine output, median [IQR] (n=322), mL^c^ | 250 [175, 350] | 250 [200. 400] | 200 [150, 300] | 250 [180. 350] | 0.068 |
| Final Pathology |  |  |  |  | 0.003 |
| *Acute cholecystitis* | 234 (36.8) | 58 (26.5) | 101 (40.6) | 75 (44.6) |  |
| *Chronic cholecystitis only* | 391 (61.5) | 156 (71.2) | 145 (58.2) | 90 (53.6) |  |
| *All other benign* | 11 (1.7) | 5 (2.3) | 3 (1.2) | 3 (1.8) |  |
| Gallstones present (pathology or intra-operatively) | 547 (86.0) | 195 (890) | 209 (83.9) | 143 (85.1) | 0.263 |

Abbreviations: IQR interquartile range.

^a^ Conversion to open cholecystectomy excluding 6 open cholecystectomies.

^b^ Excludes primary open umbilical hernia repairs through trocar site.

^c^ Not recorded in electronic medical record of all patients and minor procedures.

^d^ Operative time refers to the time the patient entered the operating room to the time the patient exited the operating room.

Table 3. Post-operative outcomes by surgery eras.

| **Characteristics** | **Total** | **Pre-robotic-assisted surgery era (%)** | **Transition period (%)** | **Robotic-assisted surgery era (%)** | **p-value** |
| --- | --- | --- | --- | --- | --- |
|  | 636 (100.0) | 219 (34.4) | 249 (39.2) | 168 (26.4) |  |
| **Post-Operative Outcomes** | | | | | |
| Received transfusion (POD 0-30 days) |  |  |  |  |  |
| *Packed red blood cells* | 15 (2.4) | 7 (3.2) | 8 (3.2) | 0 (0.0) | 0.062 |
| *Platelets* | 4 (0.6) | 2 (0.9) | 2 (0.8) | 0 (0.0) | 0.421 |
| *Plasma* | 1 (0.2) | 0 (0.0) | 1 (0.4) | 0 (0.0) | 0.459 |
| Post-operative antibiotics given | 137 (21.5) | 54 (24.7) | 54 (21.7) | 29 (17.3) | 0.214 |
| Complication (POD 0-30 days)^a^ | 41 (6.4) | 21 (9.6) | 12 (4.8) | 8 (4.8) | 0.065 |
| *Superficial SSI* | 20 (3.1) | 11 (5.0) | 4 (1.6) | 5 (3.0) | 0.105 |
| *Deep SSI* | 13 (2.0) | 6 (2.7) | 5 (2.0) | 1 (1.2) | 0.565 |
| *Retained stone or sludge* | 5 (0.8) | 3 (1.4) | 1 (0.4) | 1 (0.6) | 0.537 |
| *Biliary leak* | 10 (1.6) | 4 (1.8) | 5 (2.0) | 1 (0.6) | 0.565 |
| Length of stay, mean (SD) |  |  |  |  |  |
| *Pre-operative* | 1.0 (2.4) | 0.9 (3.0) | 1.2 (2.4) | 0.7 (1.4) | 0.003 |
| *Post-operative* | 2.2 (3.2) | 2.2 (2.5) | 2.4 (3.5) | 1.8 (3.3) | 0.013 |
| Re-admissions 30 days after discharge |  |  |  |  | 0.746 |
| *0* | 604 (95.0) | 205 (93.6) | 237 (95.2) | 162 (96.4) |  |
| *1* | 28 (4.4) | 12 (5.5) | 11 (4.4) | 5 (3.0) |  |
| *2* | 4 (0.6) | 2 (0.9) | 1 (0.4) | 1 (0.6) |  |
| ED visits 30 days after discharge |  |  |  |  | 0.540 |
| *0* | 531 (83.5) | 183 (83.6) | 205 (82.3) | 143 (85.1) |  |
| *1* | 82 (12.9) | 28 (12.8) | 37 (14.9) | 17 (12.1) |  |
| *2* | 17 (2.7) | 6 (2.7) | 5 (2.0) | 6 (3.6) |  |
| *3* | 3 (0.5) | 1 (0.5) | 2 (0.8) | 0 (0.0) |  |
| *4* | 3 (0.5) | 1 (0.5) | 0 (0.0) | 2 (1.2) |  |
| Number of additional procedures (POD 0-30 days) |  |  |  |  | 0.081 |
| *0* | 609 (95.8) | 205 (93.6) | 241 (96.8) | 163 (97.0) |  |
| *1* | 16 (2.5) | 9 (4.1) | 3 (1.2) | 4 (2.4) |  |
| *2* | 9 (1.4) | 5 (2.3) | 4 (1.6) | 0 (0.0) |  |
| *3* | 1 (0.2) | 0 (0.0) | 0 (0.0) | 1 (0.6) |  |
| *4* | 1 (0.2) | 0 (0.0) | 1 (0.4) | 0 (0.0) |  |
| Number of ERCP/EUS performed (POD 0-30 days) |  |  |  |  | 0.543 |
| *0* | 617 (97.0) | 210 (95.9) | 243 (97.6) | 164 (97.6) |  |
| *1* | 16 (2.5) | 8 (3.6) | 4 (1.6) | 4 (2.4) |  |
| *2* | 3 (0.5) | 1 (0.5) | 2 (0.8) | 0 (0.0) |  |
| Number of IR procedures performed (POD 0-30 days) |  |  |  |  | 0.368 |
| *0* | 5623 (98.0) | 212 (96.8) | 244 (98.0) | 167 (99.4) |  |
| *1* | 8 (1.3) | 5 (2.3) | 3 (1.2) | 0 (0.0) |  |
| *2* | 5 (0.8) | 2 (0.9) | 2 (0.8) | 1 (0.6) |  |
| Number of re-operations (POD 0-30 days) |  |  |  |  | 1.000 |
| *0* | 632 (99.4) | 218 (99.5) | 247 (99.2) | 167 (99.4) |  |
| *1* | 4 (0.6) | 1 (0.5) | 2 (0.8) | 1 (0.6) |  |
| Number of post-operative visits |  |  |  |  | 0.138 |
| *0* | 97 (15.2) | 39 (17.8) | 29 (11.6) | 29 (17.3) |  |
| *1* | 461 (72.5) | 147 (67.1) | 192 (77.1) | 122 (72.6) |  |
| *2* | 55 (8.6) | 23 (10.5) | 18 (7.2) | 14 (8.3) |  |
| *3* | 15 (2.4) | 4 (1.8) | 8 (3.2) | 3 (1.8) |  |
| *4* | 5 (0.8) | 3 (1.4) | 2 (0.8) | 0 (0.0) |  |
| *5* | 3 (0.5) | 3 (1.4) | 0 (0.0) | 0 (0.0) |  |
| Discharged from clinic after first visit | 463 (72.9) | 151 (69.0) | 191 (76.7) | 121 (72.5) | 0.167 |

Abbreviations: IQR interquartile range, SSI surgical site infection, SD standard deviation, ED emergency department, POD post-operative day, ERCP endoscopic retrograde cholangiopancreatography, EUS endoscopic ultrasound, IR interventional radiology.
^a^ Complication included: superficial and deep surgical site infections, biliary leak, and/or retained stone of sludge.


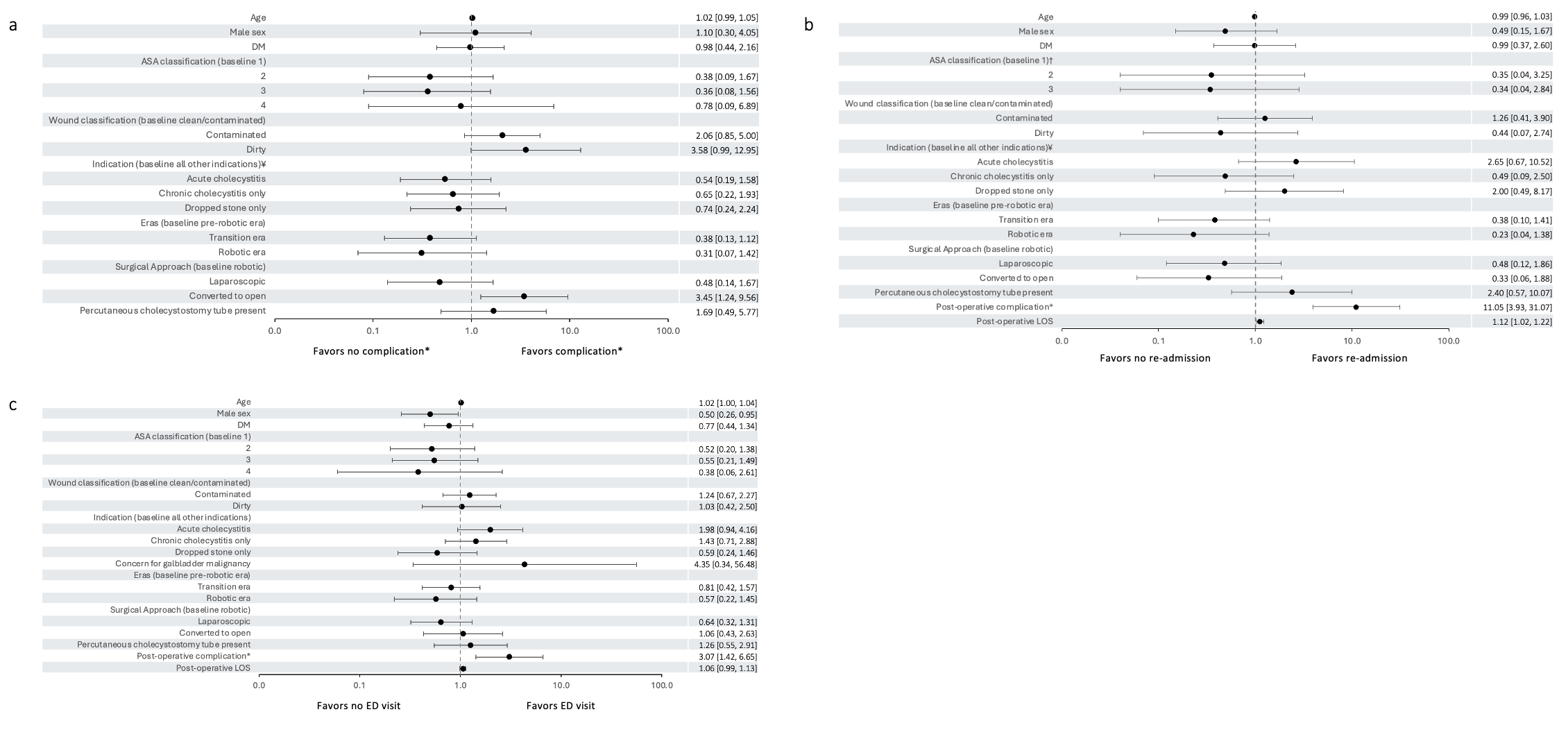


*Figure 1. Multivariable analysis of complications (a), 30-day re-admission (b), and 30-day emergency department visit (c).
¥ Concern for gallbladder malignancy omitted and 2 observations not used; * Complications include: superficial and deep surgical site infections, biliary leak, and/or retained stone of sludge; †ASA 4 omitted due to collinearity.*

Table 4. Demographic and clinical characteristics between robotic and all non-robotic cholecystectomies.

| **Characteristics** | **Total** | **Robotic-assisted cholecystectomy** | **Non-robotic-assisted cholecystectomy** | **p-value** |
| --- | --- | --- | --- | --- |
|  | 636 (100.0) | 285 (44.8) | 351 (55.2) |  |
| **Patient Characteristics** | | | | |
| Age, median [IQR] | 61 [46, 70] | 61 [45, 72] | 61 [46, 69] | 0.531 |
| Sex, male (%) | 547 (86.0) | 249 (87.4) | 298 (84.9) | 0.372 |
| Race |  |  |  | 0.495 |
| *White* | 412 (64.8) | 185 (64.9) | 227 (64.7) |  |
| *Black or African American* | 115 (18.1) | 46 (16.1) | 69 (19.7) |  |
| *Asian* | 249 (4.6) | 18 (6.3) | 11 (3.1) |  |
| *American Indian or Alaskan Native* | 3 (0.5) | 1 (0.4) | 2 (0.6) |  |
| *Native Hawaiian or other Pacific Islander* | 9 (1.4) | 5 (1.8) | 4 (1.1) |  |
| *Two or more races* | 4 (0.6) | 2 (0.7) | 2 (0.6) |  |
| *Declined or unknown or unanswered* | 64 (10.1) | 28 (9.8) | 36 (10.3) |  |
| Ethnicity |  |  |  | 0.512 |
| *Not Hispanic of Latino* | 420 (66.0) | 191 (67.0) | 229 (65.2) |  |
| *Hispanic of Latino* | 177 (27.8) | 80 (28.1) | 97 (27.6) |  |
| *Declined or unknown* | 39 (6.1) | 14 (4.9) | 25 (7.1) |  |
| History of prior surgery |  |  |  | 0.555 |
| *Abdominal* | 204 (32.1) | 96 (33.7) | 108 (30.8) |  |
| *Non-abdominal or none* | 427 (67.1) | 186 (65.3) | 241 (68.7) |  |
| *Unknown* | 5 (0.8) | 3 (1.0) | 2 (0.6) |  |
| Tobacco use^a^ | 126 (19.8) | 50 (17.5) | 76 (21.6) | 0.196 |
| Marijuana use^a^ | 63 (9.9) | 33 (11.6) | 30 (8.6) | 0.203 |
| Alcohol use disorder | 32 (5.6) | 18 (6.3) | 17 (4.8) | 0.418 |
| ASA (n=634)^b^ |  |  |  | 0.064 |
| *1* | 35 (5.5) | 14 (4.9) | 21 (6.0) |  |
| *2* | 173 (27.3) | 64 (22.5) | 109 (31.1) |  |
| *3* | 414 (65.3) | 199 (70.1) | 215 (61.4) |  |
| *4* | 12 (1.9) | 7 (2.5) | 5 (1.4) |  |
| CVD^c^ | 149 (23.4) | 71 (24.9) | 78 (22.2) | 0.426 |
| Arrythmias | 62 (9.8) | 34 (11.9) | 28 (8.0) | 0.095 |
| History of DVT or PE | 20 (3.1) | 13 (4.6) | 7 (2.0) | 0.065 |
| Respiratory Diagnosis | 240 (37.7) | 128 (44.9) | 112 (31.9) | 0.001 |
| Diabetes mellitus | 169 (26.6) | 77 (27.0) | 92 (26.2) | 0.819 |
| CVA of TIA | 32 (5.0) | 19 (6.7) | 13 (3.7) | 0.089 |
| HLD, dyslipidemia, or hypertriglyceridemia | 305 (48.0) | 153 (53.7) | 152 (43.3) | 0.009 |
| HTN | 334 (52.5) | 144 (50.5) | 190 (54.1) | 0.365 |
| Cirrhosis | 21 (3.3) | 9 (3.2) | 12 (3.4) | 0.855 |
| Liver disease excluding cirrhosis | 148 (23.3) | 71 (24.9) | 77 (21.9) | 0.377 |
| Renal disease |  |  |  | 0.075 |
| *None* | 546 (85.8) | 235 (82.5) | 311 (88.6) |  |
| *CKD* | 85 (13.4) | 47 (16.5) | 38 (10.8) |  |
| *ESRD on HD* | 5 (0.8) | 3 (1.0) | 2 (0.6) |  |
| WBC at presentation, median [IQR] (n=228), x10^9^/L^bd^ | 11.3 [8.3, 14.4] | 11.2 [8.2, 13.4] | 11.6 [8.6, 15.3] | 0.273 |
| Most recent WBC, median [IQR], x10^9^/L | 7.5 [5.9, 9.8] | 7.3 [5.8, 9.7] | 7.6 [6.1, 10.0] | 0.400 |
| A1c (all), median [IQR] (n=596), %^b^ | 5.7 [5.3, 6.3] | 5.6 [5.3, 6.3] | 5.8 [5.4, 6.3] | 0.425 |
| A1c <3 mths pre-op, median [IQR] (n=421), %^b^ | 5.7 [5.4, 6.4] | 5.6 [5.4, 6.4] | 5.7 [5.3, 6.2] | 0.879 |
| Albumin, median [IQR] (n=632), g/dL^b^ | 4.1 [3.6, 4.3] | 4.1 [3.6, 4.4] | 4 [3.6, 4.3] | 0.012 |
| Total bilirubin, median [IQR] (n=635), mg/dL^b^ | 0.8 [0.5, 1.1] | 0.7 [0.5, 1.1] | 0.8 [0.6, 1.1] | 0.078 |
| BMI, median [IQR] (n=635), kg/m^2 b^ | 29.5 [26.1, 33.0] | 28.9 [26.0, 32.8] | 29.65 [26.3, 33.3] | 0.299 |
| Surgical Indication |  |  |  | 0.126 |
| *Benign only* | 180 (28.3.2) | 66 (23.2) | 114 (32.5) |  |
| *Acute cholecystitis* | 211 (33.2) | 104 (36.5) | 107 (30.5) |  |
| *Chronic cholecystitis only* | 133 (20.9) | 64 (22.5) | 69 (19.7) |  |
| *Dropped stone only*^∆^ | 108 (17.0) | 49 (17.2) | 59 (16.8) |  |
| *Concern for gallbladder malignancy* | 4 (0.6) | 2 (0.7) | 2 (0.6) |  |

Abbreviations: IQR interquartile range, ASA American Society of Anesthesiologists classification system, CVD cardiovascular disease, DVT deep vein thrombosis, PE pulmonary embolism, CVA cerebrovascular accident, TIA transient ischemic attack, HLD hyperlipidemia, HTN hypertension, CKD chronic kidney disease, ESRD end-stage renal disease, HD hemodialysis, WBC white blood cell count, BMI body mass index

^a^ Use within 8 weeks of surgery

^b^ Not recorded in electronic medical record of all patients.

^c^ Includes heart failure, valvular and non-valvular disease, peripheral artery disease, aneurysms.

^d^ Includes disease processes with stones outside of the gallbladder such as choledocholithiasis, gallstone pancreatitis.

^e^ Only obtained if patient admitted pre-operatively (excludes outpatients).

Table 5. Operative characteristics between robotic and all non-robotic cholecystectomies.

| **Characteristics** | **Total (%)** | **Robotic cholecystectomy (%)** | **Non-robotic cholecystectomy (%)** | **p-value** |
| --- | --- | --- | --- | --- |
|  | 636 (100.0) | 285 (44.8) | 351 (55.2) |  |
| **Operative Characteristics** | | | | |
| Surgery Schedule Type |  |  |  | 0.001 |
| *Elective* | 437 (68.7) | 177 (62.1) | 260 (74.1) |  |
| *Non-elective* | 199 (31.3) | 108 (37.9) | 91 (25.9) |  |
| Surgical Technique |  |  |  | <0.001 |
| *Robotic* | 285 (44.8) | 285 (100.0) | 0 (0.0) |  |
| *Laparoscopic* | 304 (47.8) | 0 (0.0) | 304 (86.6) |  |
| *Primary Open* | 6 (0.9) | 0 (0.0) | 6 (1.7) |  |
| *Converted to open* | 41 (6.9) | 0 (0.0) | 41 (11.7) |  |
| Conversion (n=630)^a^ | 41 (6.5) | 0 (0.0) | 41 (11.9) | <0.001 |
| By novice robotic surgeon (n=285 robotic cases) | 177 (62.1) | 177 (62.1) | - | - |
| Intra-operative cholangiogram | 20 (3.1) | 2 (0.7) | 18 (5.1) | 0.001 |
| Secondary procedure performed^b^ | 90 (14.2) | 46 (16.1) | 44 (12.5) | 0.195 |
| *Percutaneous cholecystostomy removed* | 39 (6.1) | 24 (8.4) | 15 (4.3) | 0.030 |
| Subtotal performed |  |  |  | 0.582 |
| *None* | 616 (96.9) | 278 (96.9) | 338 (96.3) |  |
| *Fenestrating* | 4 (0.6) | 2 (0.7) | 2 (0.6) |  |
| *Reconstituting* | 16 (2.5) | 5 (1.8) | 11 (3.13) |  |
| Intra-operative complication | 5 (0.8) | 2 (0.7) | 3 (0.8) | 1.000 |
| Wound classification |  |  |  | 0.145 |
| *Clean* | 0 (0.0) | 0 (0.0) | 0 (0.0) |  |
| *Clean/contaminated* | 272 (42.8) | 117 (41.0) | 155 (44.2) |  |
| *Contaminated* | 284 (44.6) | 124 (43.5) | 160 (45.6) |  |
| *Dirty* | 80 (12.6) | 44 (15.4) | 36 (10.3) |  |
| Pre-operative antibiotics given | 182 (28.6) | 88 (30.9) | 94 (26.8) | 0.256 |
| Intra-operative drain placement, median [IQR] | 0 [0, 0] | 0 [0, 0] | 0 [0, 0] | 0.629 |
| Port placement, median [IQR] (n=625)^c^ | 4 [4, 4] | 4 [4, 5] | 4 [4, 4] | <0.001 |
| Operative time, median [IQR] (n=634), min^cd^ | 120 [94, 162] | 119 [98, 152] | 122 [91, 171] | 0.591 |
| Estimated blood loss, median [IQR], mL | 10 [15, 25] | 10 [5, 20] | 20 [10, 50] | <0.001 |
| Urine output, median [IQR] (n=322), mL^c^ | 250 [175, 350] | 250 [175, 350] | 240 [175, 350] | 0.307 |
| Final Pathology |  |  |  | 0.011 |
| *Acute cholecystitis* | 234 (36.8) | 123 (43.2) | 111 (31.6) |  |
| *Chronic cholecystitis only* | 391 (61.5) | 158 (55.4) | 233 (66.4) |  |
| *All other benign* | 11 (1.7) | 4 (1.4) | 7 (2.0) |  |
| Gallstones present (pathology or intra-operatively) | 547 (86.0) | 244 (85.6) | 303 (86.3) | 0.797 |

Abbreviations: IQR interquartile range.

^a^ Conversion to open cholecystectomy excluding 6 open cholecystectomies.

^b^ Excludes primary open umbilical hernia repairs through trocar site and minor procedures.

^c^ Not recorded in electronic medical record of all patients.

^d^ Operative time refers to the time the patient entered the operating room to the time the patient exited the operating room.

Table 6. Post-operative outcomes between robotic and all non-robotic cholecystectomies.

| **Characteristics** | **Total (%)** | **Robotic cholecystectomy (%)** | **Non-robotic cholecystectomy (%)** | **p-value** |
| --- | --- | --- | --- | --- |
|  | 636 (100.0) | 285 (44.8) | 351 (55.2) |  |
| **Post-Operative Outcomes** | | | | |
| Received transfusion (POD 0-30 days) |  |  |  |  |
| *Packed red blood cells* | 15 (2.4) | 3 (1.0) | 12 (3.4) | 0.051 |
| *Platelets* | 4 (0.6) | 1 (0.4) | 3 (0.8) | 0.424 |
| *Plasma* | 1 (0.2) | 1 (0.4) | 0 (0.0) | 0.267 |
| Post-operative antibiotics given | 137 (21.5) | 53 (18.6) | 84 (23.9) | 0.104 |
| Complication (POD 0-30 days)^a^ | 41 (6.4) | 15 (5.3) | 26 (7.4) | 0.273 |
| *Superficial SSI* | 20 (3.1) | 7 (2.5) | 13 (3.7) | 0.370 |
| *Deep SSI* | 13 (2.0) | 5 (1.8) | 8 (2.3) | 0.642 |
| *Retained stone or sludge* | 5 (0.8) | 1 (0.4) | 4 (1.1) | 0.387 |
| *Biliary leak* | 10 (1.6) | 5 (1.8) | 5 (1.4) | 0.739 |
| Length of stay, mean (SD) |  |  |  |  |
| *Pre-operative* | 1.0 (2.4) | 0.8 (1.6) | 1.1 (2.9) | 0.490 |
| *Post-operative* | 2.2 (3.2) | 1.9 (3.0) | 2.4 (3.3) | 0.018 |
| Re-admissions 30 days after discharge, median [IQR] |  |  |  | 0.889 |
| *0* | 604 (95.0) | 272 (95.4) | 332 (94.6) |  |
| *1* | 28 (4.4) | 11 (3.9) | 17 (4.8) |  |
| *2* | 4 (0.6) | 2 (0.7) | 2 (0.6) |  |
| ED visits 30 days after discharge, median |  |  |  | 0.885 |
| *0* | 531 (83.5) | 237 (83.2) | 294 (83.8) |  |
| *1* | 82 (12.9) | 36 (12.6) | 46 (13.1) |  |
| *2* | 17 (2.7) | 8 (2.8) | 9 (2.6) |  |
| *3* | 3 (0.5) | 2 (0.7) | 1 (0.3) |  |
| *4* | 3 (0.5) | 2 (0.7) | 1 (0.3) |  |
| Number of additional procedures (POD 0-30 days) |  |  |  | 0.573 |
| *0* | 609 (95.8) | 275 (96.5) | 334 (95.2) |  |
| *1* | 16 (2.5) | 5 (1.8) | 11 (3.1) |  |
| *2* | 9 (1.4) | 4 (1.4) | 5 (1.4) |  |
| *3* | 1 (0.2) | 1 (0.4) | 0 (0.0) |  |
| *4* | 1 (0.2) | 0 (0.0) | 1 (0.3) |  |
| Number of ERCP/EUS performed (POD 0-30 days) |  |  |  | 1.000 |
| *0* | 617 (97.0) | 277 (97.2) | 340 (96.9) |  |
| *1* | 16 (2.5) | 7 (2.5) | 9 (2.6) |  |
| *2* | 3 (0.5) | 1 (0.4) | 2 (0.6) |  |
| Number of IR procedures performed (POD 0-30 days) |  |  |  | 0.567 |
| *0* | 5623 (98.0) | 281 (98.6) | 342 (97.4) |  |
| *1* | 8 (1.3) | 2 (0.7) | 6 (1.7) |  |
| *2* | 5 (0.8) | 2 (0.7) | 3 (0.9) |  |
| Number of re-operations (POD 0-30 days) |  |  |  | 1.000 |
| *0* | 632 (99.4) | 283 (99.3) | 349 (99.4) |  |
| *1* | 4 (0.6) | 2 (0.7) | 2 (0.6) |  |
| Number of post-operative visits |  |  |  | 0.115 |
| *0* | 97 (15.2) | 41 (14.4) | 56 (16.0) |  |
| *1* | 461 (72.5) | 213 (74.7) | 248 (70.7) |  |
| *2* | 55 (8.6) | 22 (7.7) | 33 (9.4) |  |
| *3* | 15 (2.4) | 9 (3.2) | 6 (1.7) |  |
| *4* | 5 (0.8) | 0 (0.0) | 5 (1.4) |  |
| *5* | 3 (0.5) | 0 (0.0) | 3 (0.8) |  |
| Discharged from clinic after first visit | 463 (72.9) | 212 (74.6) | 251 (71.5) | 0.376 |

Abbreviations: IQR interquartile range, SSI surgical site infection, SD standard deviation, ED emergency department, POD post-operative day, ERCP endoscopic retrograde cholangiopancreatography, EUS endoscopic ultrasound, IR interventional radiology.
^a^ Complication included: superficial and deep surgical site infections, biliary leak, and/or retained stone of sludge.

Table 7. Demographic and clinical characteristics of patients pre-operatively diagnosed with acute cholecystitis.

|  | **Patients, with pre-operative diagnosis of acute cholecystitis** | | | |
| --- | --- | --- | --- | --- |
| **Characteristics** | **Total** | **Robotic cholecystectomy** | **Non-robotic cholecystectomy** | **p-value** |
|  | 211 (100.0) | 104 (49.3) | 107 (50.7) |  |
| **Patient Characteristics** | | | | |
| Age, median [IQR] | 62 [53, 72] | 61 [51, 74] | 64 [55, 71] | 0.986 |
| Sex, male | 181 (85.8) | 89 (85.6) | 92 (86.0) | 0.933 |
| Race |  |  |  | 0.264 |
| *White* | 130 (61.6) | 66 (63.5) | 64 (59.8) |  |
| *Black or African American* | 49 (23.2) | 22 (21.2) | 27 (25.2) |  |
| *Asian* | 7 (3.3) | 6 (5.8) | 1 (0.9) |  |
| *American Indian or Alaskan Native* | 0 (0.0) | 0 (0.0) | 0 (0.0) |  |
| *Native Hawaiian or other Pacific Islander* | 3 (1.4) | 1 (1.0) | 2 (1.9) |  |
| *Two or more races* | 2 (1.0) | 0 (0.0) | 2 (1.9) |  |
| *Unknown* | 20 (9.5) | 9 (8.6) | 11 (10.3) |  |
| Ethnicity |  |  |  | 0.957 |
| *Not Hispanic of Latino* | 142 (67.3) | 69 (66.4) | 73 (68.2) |  |
| *Hispanic of Latino* | 55 (26.1) | 28 (26.9) | 27 (25.23) |  |
| *Declined or unknown* | 14 (6.6) | 7 (6.7) | 7 (6.5) |  |
| History of prior surgery |  |  |  | 0.492 |
| *Abdominal* | 70 (33.2) | 36 (34.6) | 34 (31.8) |  |
| *Non-abdominal or none* | 137 (64.9) | 65 (62.5) | 72 (67.3) |  |
| *Unknown* | 4 (1.9) | 3 (2.9) | 1 (0.9) |  |
| Tobacco use^a^ | 33 (15.6) | 16 (15.4) | 17 (15.9) | 0.920 |
| Marijuana use^a^ | 16 (7.6) | 7 (6.7) | 9 (8.4) | 0.645 |
| Alcohol use disorder | 15 (7.1) | 10 (9.6) | 5 (4.7) | 0.162 |
| ASA (n=185)^b^ |  |  |  | 0.578 |
| *1* | 17 (8.1) | 8 (7.7) | 9 (8.5) |  |
| *2* | 61 (29.0) | 27 (26.0) | 34 (32.1) |  |
| *3* | 128 (61.0) | 66 (63.5) | 62 (58.5) |  |
| *4* | 4 (1.9) | 3 (2.9) | 1 (0.9) |  |
| CVD^c^ | 54 (25.6) | 28 (26.9) | 26 (24.3) | 0.662 |
| Arrythmias | 17 (8.1) | 10 (9.6) | 7 (6.5) | 0.412 |
| History of DVT or PE | 5 (2.4) | 2 (1.9) | 3 (2.8) | 1.000 |
| Respiratory Diagnosis | 79 (37.4) | 46 (44.2) | 33 (30.8) | 0.045 |
| Diabetes mellitus | 56 (26.5) | 33 (31.7) | 23 (21.5) | 0.092 |
| CVA | 10 (4.7) | 6 (5.8) | 4 (3.7) | 0.534 |
| HLD, dyslipidemia, or hypertriglyceridemia | 101 (47.9) | 51 (49.0) | 50 (46.7) | 0.737 |
| HTN | 111 (52.6) | 58 (55.8) | 53 (49.5) | 0.364 |
| Cirrhosis | 4 (1.9) | 1 (1.0) | 3 (2.8) | 0.622 |
| Liver disease excluding cirrhosis | 33 (15.6) | 15 (14.4) | 18 (16.8) | 0.631 |
| Renal disease |  |  |  | 0.747 |
| *None* | 188 (89.1) | 91 (87.5) | 97 (90.6) |  |
| *CKD* | 21 (10.0) | 12 (11.5) | 9 (8.4) |  |
| *ESRD on HD* | 2 (1.0) | 1 (1.0) | 1 (0.9) |  |
| WBC at presentation, median [IQR] (n=165), x10^9^/L^bd^ | 12.2 [9.3, 16.1] | 11.6 [9.0, 14.6] | 13.4 [10.1, 16.5] | 0.081 |
| Most recent WBC, median [IQR], x10^9^/L | 9.8 [6.6, 13.8] | 9.8 [6.7, 12.9] | 10.2 [6.6, 14.0] | 0.834 |
| A1c (all), median [IQR] (n=188), %^b^ | 5.8 [5.4, 6.3] | 5.8 [5.4, 6.5] | 5.8 [5.3, 6.2] | 0.182 |
| A1c <3 mths pre-op, median [IQR] (n=105), %^b^ | 5.8 [5.4, 6.5] | 6.1 [5.4, 7.0] | 5.8 [5.3, 6.1] | 0.041 |
| Albumin, median [IQR] (n=210), g/dL^b^ | 3.7 [3.3, 4.2] | 3.7 [3.4, 4.2] | 3.7 [3.2, 4.1] | 0.173 |
| Total bilirubin, median [IQR], mg/dL | 0.9 [0.6, 1.3] | 0.7 [0.5, 1.2] | 1.0 [0.7, 1.3] | 0.034 |
| BMI, median [IQR], kg/m^2^ | 28.8 [25.9, 32.9] | 28.4 [25.4, 31.9] | 30.3 [26.5, 33.4] | 0.071 |

Abbreviations: IQR interquartile range, ASA American Society of Anesthesiologists classification system, CVD cardiovascular disease, DVT deep vein thrombosis, PE pulmonary embolism, CVA cerebrovascular accident, TIA transient ischemic attack, HLD hyperlipidemia, HTN hypertension, CKD chronic kidney disease, ESRD end-stage renal disease, HD hemodialysis, WBC white blood cell count, BMI body mass index

^a^ Use within 8 weeks of surgery

^b^ Not recorded in electronic medical record of all patients.

^c^ Includes heart failure, valvular and non-valvular disease, peripheral artery disease, aneurysms.

^d^ Only obtained if patient admitted pre-operatively (excludes outpatients).

Table 8. Operative characteristics in patients pre-operatively diagnosed with acute cholecystitis.

|  | **Patients, with pre-operative diagnosis of acute cholecystitis** | | | |
| --- | --- | --- | --- | --- |
| **Characteristics** | **Total (%)** | **Robotic cholecystectomy (%)** | **Non-robotic cholecystectomy (%)** | **p-value** |
|  | 211 (100.0) | 104 (49.3) | 107 (50.7) |  |
| **Operative Characteristics** | | | | |
| Surgery Schedule Type |  |  |  | 0.036 |
| *Elective* | 65 (30.8) | 25 (24.0) | 40 (37.4) |  |
| *Non-elective* | 146 (69.2) | 79 (76.0) | 67 (62.2) |  |
| Surgical Technique |  |  |  | <0.001 |
| *Robotic* | 104 (49.3) | 104 (100.0) | 0 (0.0) |  |
| *Laparoscopic* | 80 (37.9) | 0 (0.0) | 80 (74.8) |  |
| *Primary Open* | 2 (1.0) | 0 (0.0) | 2 (1.1) |  |
| *Converted to open* | 25 (11.8) | 0 (0.0) | 25 (23.4) |  |
| Conversion (n=209)^a^ | 25 (12.0) | 0 (0.0) | 25 (23.8) | <0.001 |
| By novice robotic surgeon (n=104 robotic cases) | 61 (28.9) | 61 (58.6) | - | - |
| Secondary procedure performed^b^ | 40 (19.0) | 22 (21.2) | 18 (16.8) | 0.422 |
| *Percutaneous cholecystostomy removed* | 20 (9.5) | 13 (12.5) | 7 (6.5) | 0.140 |
| Intra-operative cholangiogram | 6 (2.8) | 1 (1.0) | 5 (4.7) | 0.212 |
| Subtotal performed |  |  |  | 0.173 |
| *None* | 196 (92.9) | 100 (96.2) | 96 (89.7) |  |
| *Fenestrating* | 3 (1.4) | 1 (1.0) | 2 (1.9) |  |
| *Reconstituting* | 12 (5.7) | 3 (2.9) | 9 (8.4) |  |
| Intra-operative complication, yes | 2 (1.0) | 0 (0.0) | 2 (1.9) | 0.498 |
| Wound classification |  |  |  | 0.655 |
| *Clean* | 0 (0.0) | 0 (0.0) | 0 (0.0) |  |
| *Clean/Contaminated* | 7 (3.3) | 3 (2.9) | 4 (3.7) |  |
| *Contaminated* | 134 (63.5) | 63 (60.6) | 71 (66.4) |  |
| *Dirty* | 70 (33.2) | 38 (36.5) | 32 (29.9) |  |
| Peri-operative antibiotics given | 211 (100.0) | 104 (100.0) | 107 (100.0) | - |
| Pre-operative antibiotics given | 157 (74.4) | 81 (77.9) | 76 (71.0) | 0.254 |
| Intra-operative drain placement, median [IQR] | 0 [0, 1] | 0 [0, 1] | 0 [0, 1] | 0.468 |
| Port placement, median [IQR] (n=207)^c^ | 4 [4, 4] | 4 [4, 5] | 4 [4, 4] | <0.001 |
| Operative time, median [IQR] (n=210), min^cd^ | 139 [107, 189] | 125 [106, 168] | 157.5 [109, 201] | 0.010 |
| Estimated blood loss, median [IQR], mL | 20 [10, 50] | 15 [10, 25] | 50 [20, 100] | <0.001 |
| Urine output, median [IQR] (n=118), mL^c^ | 250 [175, 350] | 250 [175, 350] | 250 [175, 350] | 0.842 |
| Final Pathology |  |  |  | 0.082 |
| *Acute (any) cholecystitis* | 166 (78.7) | 87 (83.6) | 79 (73.8) |  |
| *Chronic cholecystitis* | 45 (21.3) | 17 (16.4) | 28 (26.2) |  |
| Stone present (pathology or intra-operatively) | 182 (86.3) | 88 (84.6) | 94 (87.8) | 0.495 |

Abbreviations: IQR interquartile range.

^a^ Conversion to open cholecystectomy excluding two open cholecystectomies.

^b^ Excludes primary open umbilical hernia repairs through trocar site.

^c^ Not recorded in electronic medical record of all patients and minor procedures.

^d^ Operative time refers to the time the patient entered the operating room to the time the patient exited the operating room.

Table 9. Post-operative outcomes in patients pre-operatively diagnosed with acute cholecystitis.

|  | **Patients, with pre-operative diagnosis of acute cholecystitis** | | | |
| --- | --- | --- | --- | --- |
| **Characteristics** | **Total (%)** | **Robotic cholecystectomy (%)** | **Non-robotic cholecystectomy (%)** | **p-value** |
|  | 211 (100.0) | 104 (49.3) | 107 (50.7) |  |
| **Post-Operative Outcomes** | | | | |
| Received transfusion (POD 0-30 days) |  |  |  |  |
| *Packed red blood cells* | 8 (3.8) | 2 (1.9) | 6 (5.6) | 0.280 |
| *Platelets* | 2 (1.0) | 0 (0.0) | 2 (1.9) | 0.498 |
| *Plasma* | 1 (0.5) | 1 (1.0) | 0 (0.0) | 0.493 |
| Post-operative antibiotics given | 87 (41.2) | 31 (29.8) | 56 (52.3) | 0.001 |
| Complication (POD 0-30 days)^a^ | 16 (7.6) | 5 (4.8) | 11 (10.3) | 0.133 |
| *Superficial SSI* | 6 (2.8) | 1 (1.0) | 5 (4.7) | 0.212 |
| *Deep SSI* | 6 (2.8) | 2 (1.9) | 4 (3.7) | 0.683 |
| *Retained stone or sludge* | 1 (0.5) | 0 (0.0) | 1 (0.9) | 1.000 |
| *Biliary leak* | 4 (1.9) | 2 (1.9) | 2 (1.9) | 1.000 |
| Length of stay, mean (SD) |  |  |  |  |
| *Pre-operative* | 1.5 (2.2) | 1.2 (1.6) | 1.8 (2.6) | 0.179 |
| *Post-operative* | 3.2 (4.2) | 2.4 (3.2) | 4.1 (4.8) | 0.001 |
| Re-admissions 30 days after discharge |  |  |  | 0.896 |
| *0* | 195 (92.4) | 96 (92.3) | 99 (92.5) |  |
| *1* | 15 (7.1) | 7 (6.7) | 8 (7.5) |  |
| *2* | 1 (0.5) | 1 (1.0) | 0 (0.0) |  |
| ED visits 30 days after discharge |  |  |  | 0.897 |
| *0* | 159 (75.4) | 81 (77.9) | 78 (72.9) |  |
| *1* | 39 (18.5) | 18 (17.3) | 21 (19.6) |  |
| *2* | 9 (4.3) | 3 (2.9) | 6 (5.6) |  |
| *3* | 2 (1.0) | 1 (1.0) | 1 (0.9) |  |
| *4* | 2 (1.0) | 1 (1.0) | 1 (0.9) |  |
| Number of additional procedures (POD 0-30 days) |  |  |  | 0.939 |
| *0* | 202 (95.7) | 100 (96.2) | 102 (95.3) |  |
| *1* | 5 (2.4) | 2 (1.9) | 3 (2.8) |  |
| *2* | 3 (1.4) | 2 (1.9) | 1 (0.9) |  |
| *3* | 0 (0.0) | 0 (0.0) | 0 (0.0) |  |
| *4* | 1 (0.5) | 0 (0.0) | 1 (0.9) |  |
| Number of ERCP/EUS performed (POD 0-30 days) |  |  |  | 1.000 |
| *0* | 205 (97.2) | 101 (97.1) | 104 (97.2) |  |
| *1* | 4 (1.9) | 2 (1.9) | 2 (1.9) |  |
| *2* | 2 (1.0) | 1 (1.0) | 1 (0.9) |  |
| Number of IR procedures performed (POD 0-30 days) |  |  |  | 1.000 |
| *0* | 207 (98.1) | 103 (99.0) | 104 (97.2) |  |
| *1* | 1 (0.5) | 0 (0.0) | 1 (0.9) |  |
| *2* | 3 (1.4) | 1 (1.0) | 2 (1.9) |  |
| Number of re-operations (POD 0-30 days) |  |  |  | 1.000 |
| *0* | 210 (99.5) | 104 (100.0) | 106 (99.1) |  |
| *1* | 1 (0.5) | 0 (0.0) | 1 (0.9) |  |
| Number of post-operative visits |  |  |  | 0.164 |
| *0* | 28 (13.3) | 15 (14.4) | 13 (12.2) |  |
| *1* | 151 (71.6) | 77 (74.0) | 74 (69.2) |  |
| *2* | 23 (10.9) | 8 (7.7) | 15 (14.0) |  |
| *3* | 5 (2.4) | 4 (3.8) | 1 (0.9) |  |
| *4* | 2 (1.0) | 0 (0.0) | 2 (1.9) |  |
| *5* | 2 (1.0) | 0 (0.0) | 2 (1.9) |  |
| Discharged from clinic after first visit | 156 (74.3) | 78 (78.7) | 78 (72.9) | 0.639 |

Abbreviations: IQR interquartile range, SSI surgical site infection, SD standard deviation, ED emergency department, POD post-operative day, ERCP endoscopic retrograde cholangiopancreatography, EUS endoscopic ultrasound, IR interventional radiology.
^a^ Complications include: superficial and deep surgical site infections, biliary leak, and/or retained stone of sludge.


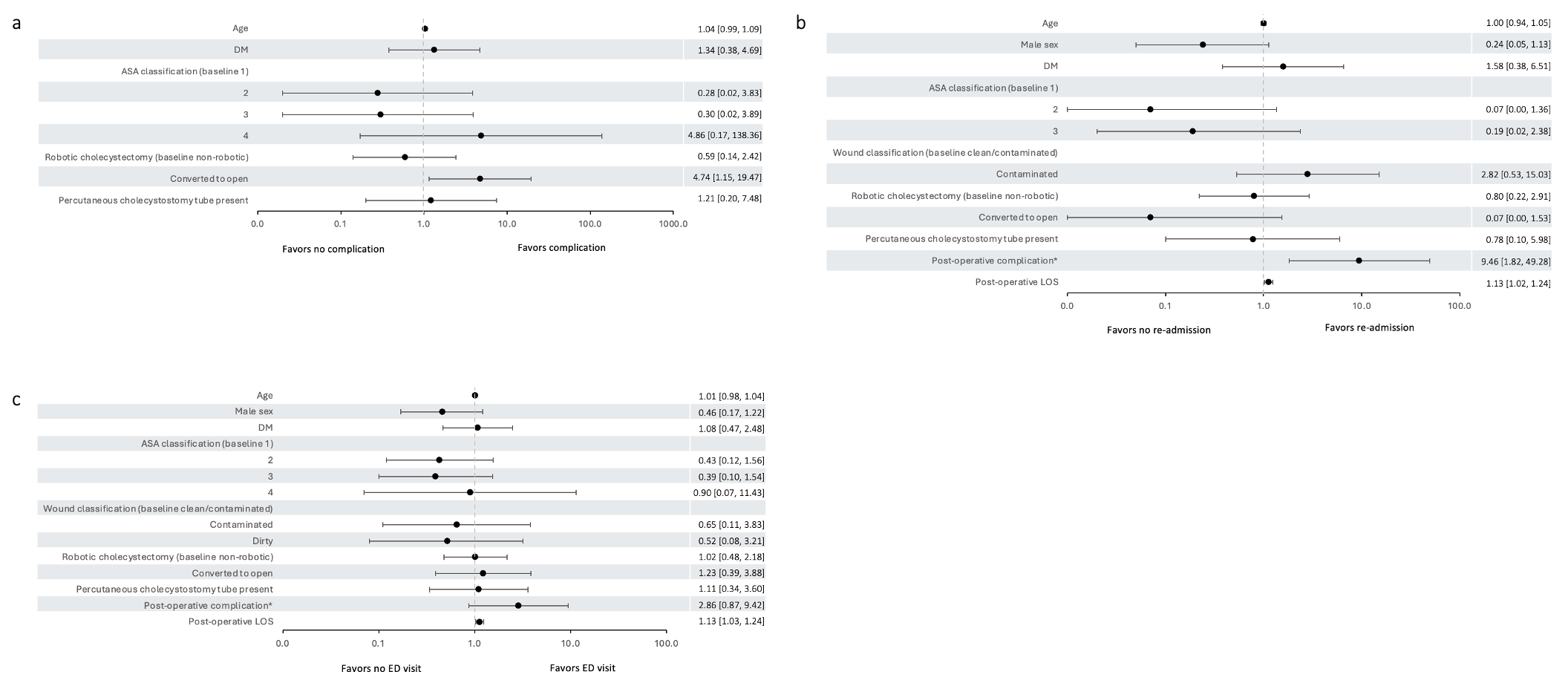


Figure 2. Multivariable analysis of complications (a), 30-day re-admission (b), and 30-day emergency department visit (c) for acute cholecystitis subgroup.
* Complications included: superficial and deep surgical site infections, biliary leak, and/or retained stone of sludge.

Table 10. Demographic and clinical characteristics of patients with a percutaneous cholecystostomy tube.

|  | **Patients, with percutaneous cholecystostomy tube placement at the time of surgery** | | | |
| --- | --- | --- | --- | --- |
| **Characteristics** | **Total (%)** | **Robotic cholecystectomy (%)** | **Non-robotic cholecystectomy (%)** | **p-value** |
|  | 39 (100.0) | 24 (61.5) | 15 (38.5) |  |
| **Patient Characteristics** | | | | |
| Age, median [IQR] | 71 [63, 78] | 72 [61, 79] | 68 [63, 71] | 0.525 |
| Sex, male | 37 (94.9) | 22 (91.7) | 15 (100.0) | 0.514 |
| Race |  |  |  | 0.590 |
| *White* | 23 (59.0) | 15 (62.5) | 8 (53.3) |  |
| *Black or African American* | 11 (28.2) | 7 (29.2) | 4 (26.7) |  |
| *Asian* | 1 (2.6) | 0 (0.0) | 1 (6.7) |  |
| *Native Hawaiian or other Pacific Islander* | 1 (2.6) | 0 (0.0) | 1 (6.7) |  |
| *Declined or unknown or unanswered* | 3 (7.7) | 2 (8.3) | 1 (6.7) |  |
| Ethnicity |  |  |  | 0.511 |
| *Not Hispanic of Latino* | 33 (84.6) | 19 (79.2) | 14 (93.3) |  |
| *Hispanic of Latino* | 4 (10.3) | 3 (12.5) | 1 (6.7) |  |
| *Declined or unknown* | 2 (5.1) | 2 (8.3) | 0 (0.0) |  |
| History of prior surgery |  |  |  | 0.792 |
| *Abdominal* | 14 (35.9) | 9 (37.5) | 5 (33.3) |  |
| *Non-abdominal or none* | 25 (64.1) | 15 (62.5) | 10 (66.7) |  |
| Tobacco use^a^ | 6 (15.4) | 2 (8.3) | 4 (26.7) | 0.180 |
| Marijuana use^a^ | 5 (12.8) | 2 (8.3) | 3 (20.0) | 0.354 |
| Alcohol use disorder | 2 (5.1) | 2 (8.3) | 0 (0.0) | 0.514 |
| ASA |  |  |  | 0.258 |
| *1* | 0 (0.0) | 0 (0.0) | 0 (0.0) |  |
| *2* | 5 (12.8) | 3 (12.5) | 2 (13.3) |  |
| *3* | 32 (82.0) | 21 (87.5) | 11 (73.3) |  |
| *4* | 2 (5.1) | 0 (0.0) | 2 (13.3) |  |
| CVD^b^ | 22 (56.4) | 14 (58.3) | 8 (53.3) | 0.759 |
| Arrythmias | 9 (23.1) | 9 (37.5) | 0 (0.0) | 0.007 |
| History of DVT or PE | 3 (7.7) | 3 (12.5) | 0 (0.0) | 0.271 |
| Respiratory Diagnosis | 18 (46.2) | 11 (45.8) | 7 (46.7) | 0.959 |
| Diabetes mellitus | 18 (46.2) | 10 (41.7) | 8 (53.3) | 0.477 |
| CVA of TIA | 7 (18.0) | 4 (16.7) | 3 (20.0) | 1.000 |
| HLD, dyslipidemia, or hypertriglyceridemia | 22 (56.4) | 17 (70.8) | 5 (33.3) | 0.022 |
| HTN | 31 (79.5) | 30 (83.3) | 11 (73.3) | 0.686 |
| Cirrhosis | 4 (10.3) | 1 (4.2) | 3 (20.0) | 0.279 |
| Liver disease excluding cirrhosis | 7 (18.0) | 4 (16.7) | 3 (20.0) | 1.000 |
| Renal disease |  |  |  | 0.655 |
| *None* | 30 (76.9) | 17 (70.8) | 13 (86.7) |  |
| *CKD* | 8 (20.5) | 6 (25.0) | 2 (13.3) |  |
| *ESRD on HD* | 1 (2.6) | 1 (4.2) | 0 (0.0) |  |
| WBC at presentation, median [IQR] (n=4), x10^9^/L^cd^ | 7.6 [3.8, 10.9] | 10.6 [4.6, 11.2] | 3.0 [3.0, 3.0] | 0.500 |
| Most recent WBC, median [IQR], x10^9^/L | 7.0 [5.0, 9.0] | 7.1 [5.0, 8.7] | 6.6 [3.3, 9.0] | 0.663 |
| A1c (all), median [IQR] (n=38), %^c^ | 5.8 [5.1, 6.7] | 5.6 [5.2, 6.5] | 6.1 [5.0, 6.8] | 0.934 |
| A1c <3 mths pre-op, median [IQR] (n=33), %^c^ | 5.9 [5.1, 6.7] | 5.9 [5.4, 6.7] | 5.6 [5.0, 6.7] | 0.488 |
| Albumin, median [IQR] (n=38), g/dL^c^ | 4.0 [3.6, 4.1] | 4.0 [3.4, 4.1] | 3.9 [3.6, 4.4] | 0.780 |
| Total bilirubin, median [IQR] (n=38), mg/dL^c^ | 0.7 [0.5, 0.9] | 0.6 [0.5, 0.9] | 0.8 [0.6, 1.0] | 0.324 |
| BMI, median [IQR], kg/m^2^ | 27.4 [24.2, 30.8] | 27.3 [24.1, 31.3] | 27.7 [24.2, 30.8] | 0.723 |
| Surgical Indication |  |  |  | 0.648 |
| *Acute cholecystitis* | 20 (51.3) | 13 (54.2) | 7 (46.7) |  |
| *Chronic cholecystitis only* | 19 (48.7) | 11 (45.8) | 8 (53.3) |  |

Abbreviations: IQR interquartile range, ASA American Society of Anesthesiologists classification system, CVD cardiovascular disease, DVT deep vein thrombosis, PE pulmonary embolism, CVA cerebrovascular accident, TIA transient ischemic attack, HLD hyperlipidemia, HTN hypertension, CKD chronic kidney disease, ESRD end-stage renal disease, HD hemodialysis, WBC white blood cell count, BMI body mass index

* Use within 8 weeks of surgery

† Includes heart failure, valvular and non-valvular disease, peripheral artery disease, aneurysms.

^∆^ Includes disease processes with stones outside of the gallbladder such as choledocholithiasis, gallstone pancreatitis.

** Only obtained if patient admitted pre-operatively (excludes outpatients).

^a^ Use within 8 weeks of surgery

^b^ Includes heart failure, valvular and non-valvular disease, peripheral artery disease, aneurysms.

^c^ Not recorded in electronic medical record of all patients.

^d^ Only obtained if patient admitted pre-operatively (excludes outpatients).

Table 11. Operative characteristics in patients with a percutaneous cholecystostomy tube.

|  | **Patients, with percutaneous cholecystostomy tube placement at the time of surgery** | | | |
| --- | --- | --- | --- | --- |
| **Characteristics** | **Total (%)** | **Robotic cholecystectomy (%)** | **Non-robotic cholecystectomy (%)** | **p-value** |
|  | 39 (100.0) | 24 (61.5) | 15 (38.5) |  |
| **Operative Characteristics** | | | | |
| Surgery Schedule Type |  |  |  | 1.000 |
| *Elective* | 35 (89.7) | 21 (87.5) | 14 (93.3) |  |
| *Non-elective* | 4 (10.3) | 3 (12.5) | 1 (6.7) |  |
| Surgical Technique |  |  |  | <0.001 |
| *Robotic* | 24 (61.5) | 24 (100.0) | 0 (0.0) |  |
| *Laparoscopic* | 11 (28.2) | 0 (0.0) | 11 (73.3) |  |
| *Primary Open* | 1 (2.6) | 0 (0.0) | 1 (6.7) |  |
| *Laparoscopic-converted-to-open* | 3 (7.7) | 0 (0.0) | 3 (20.0) |  |
| Conversion (n=38)^a^ | 3 (7.9) | 0 (0.0) | 3 (21.4) | 0.043 |
| Intra-operative cholangiogram | 0 (0.0) | 0 (0.0) | 0 (0.0) | - |
| By novice robotic surgeon (n=24 robotic cases) | 10 (41.7) | 10 (41.7) | - | - |
| Subtotal performed |  |  |  | 0.142 |
| *None* | 35 (89.7) | 21 (87.5) | 14 (93.3) |  |
| *Fenestrating* | 1 (2.6) | 0 (0.0) | 1 (6.7) |  |
| *Reconstituting* | 3 (7.7) | 3 (12.5) | 0 (0.0) |  |
| Intra-operative complication | 0 (0.0) | 0 (0.0) | 0 (0.0) | - |
| Wound classification |  |  |  | 0.658 |
| *Clean* | 0 (0.0) | 0 (0.0) | 0 (0.0) |  |
| *Clean/Contaminated* | 13 (33.3) | 7 (29.2) | 6 (40.0) |  |
| *Contaminated* | 21 (53.8) | 13 (54.2) | 8 (53.3) |  |
| *Dirty* | 5 (12.8) | 4 (16.7) | 1 (6.7) |  |
| Peri-operative antibiotics given | 39 (100.0) | 24 (100.0) | 15 (100.0) | - |
| Pre-operative antibiotics given | 4 (10.3) | 2 (8.3) | 2 (13.3) | 0.631 |
| Intra-operative drain placement, median [IQR] | 0 [0, 1] | 0 [0, 1] | 0 [0, 0] | 0.098 |
| Port placement, median [IQR] (n=38)^b^ | 4 [4, 5] | 5 [4, 5] | 4 [4, 4] | 0.030 |
| Operative time, median [IQR], min^c^ | 164 [105, 229] | 130 [102, 178] | 229 [154, 284] | <0.001 |
| Estimated blood loss, median [IQR], mL | 15 [10, 30] | 12 [5, 20] | 25 [20, 100] | 0.002 |
| Urine output, median [IQR] (n=22), mL^b^ | 300 [250, 400] | 350 [260, 400] | 260 [200, 400] | 0.864 |
| Final Pathology |  |  |  | 0.131 |
| *Acute cholecystitis* | 24 (61.5) | 17 (70.8) | 7 (46.7) |  |
| *Chronic cholecystitis only* | 15 (38.5) | 7 (29.2) | 8 (53.3) |  |
| Gallstones present (pathology or intra-operatively) | 28 (71.8) | 18 (75.0) | 10 (66.7) | 0.718 |

Abbreviations: IQR interquartile range.

† Excludes primary open umbilical hernia repairs through trocar site.

** Operative time refers to the time the patient entered the operating room to the time the patient exited the operating room.

^a^ Conversion to open cholecystectomy excluding one open cholecystectomy.

^b^ Not recorded in electronic medical record of all patients.

^c^ Operative time refers to the time the patient entered the operating room to the time the patient exited the operating room.

Table 12. Post-operative outcomes in patients with a percutaneous cholecystostomy tube.

|  | **Patients, with percutaneous cholecystostomy tube placement at the time of surgery** | | | |
| --- | --- | --- | --- | --- |
| **Characteristics** | **Total (%)** | **Robotic cholecystectomy (%)** | **Non-robotic cholecystectomy (%)** | **p-value** |
|  | 39 (100.0) | 24 (61.5) | 15 (38.5) |  |
| **Post-Operative Outcomes** | | | | |
| Received transfusion (POD 0-30 days) |  |  |  |  |
| *Packed red blood cells* | 3 (7.7) | 0 (0.0) | 3 (20.0) | 0.023 |
| *Platelets* | 1 (2.6) | 0 (0.0) | 1 (6.7) | 0.200 |
| *Plasma* | 0 (0.0) | 0 (0.0) | 0 (0.0) | - |
| Post-operative antibiotics given | 12 (30.8) | 4 (16.7) | 8 (53.3) | 0.031 |
| Complication (POD 0-30 days)^a^ | 4 (10.3) | 1 (4.2) | 3 (20.0) | 0.279 |
| *Superficial SSI* | 3 (7.7) | 0 (0.0) | 3 (20.0) | 0.050 |
| *Deep SSI* | 1 (2.6) | 1 (4.2) | 0 (0.0) | 1.000 |
| *Retained stone or sludge* | 0 (0.0) | 0 (0.0) | 0 (0.0) | - |
| *Biliary leak* | 1 (2.6) | 1 (4.2) | 0 (0.0) | 1.000 |
| Length of stay, mean (SD) |  |  |  |  |
| *Pre-operative* | 0.3 (1.2) | 0.4 (1.5) | 0.1 (0.2) | 0.511 |
| *Post-operative* | 2.4 (2.1) | 1.8 (1.4) | 3.5 (2.5) | 0.014 |
| Re-admissions 30 days after discharge, median [IQR] |  |  |  | 0.017 |
| *0* | 35 (89.7) | 24 (100.0) | 11 (73.3) |  |
| *1* | 3 (7.7) | 0 (0.0) | 3 (20.0) |  |
| *2* | 1 (2.6) | 0 (0.0) | 1 (6.7) |  |
| ED visits 30 days after discharge, median |  |  |  | 0.331 |
| *0* | 29 (74.4) | 19 (79.2) | 10 (66.7) |  |
| *1* | 8 (20.5) | 5 (20.8) | 3 (20.0) |  |
| *2* | 1 (2.6) | 0 (0.0) | 1 (6.7) |  |
| *3* | 1 (2.6) | 0 (0.0) | 1 (6.7) |  |
| Number of additional procedures (POD 0-30 days) |  |  |  | 0.050 |
| *0* | 35 (89.7) | 23 (95.8) | 12 (80.0) |  |
| *1* | 3 (7.7) | 0 (0.0) | 3 (20.0) |  |
| *2* | 1 (2.6) | 1 (4.2) | 0 (0.0) |  |
| Number of ERCP/EUS performed (POD 0-30 days) |  |  |  | 0.547 |
| *0* | 36 (92.3) | 23 (95.8) | 13 (86.7) |  |
| *1* | 3 (7.7) | 1 (4.2) | 2 (13.3) |  |
| Number of IR procedures performed (POD 0-30 days) |  |  |  | 1.000 |
| *0* | 37 (94.9) | 23 (95.8) | 14 (93.3) |  |
| *1* | 2 (5.1) | 1 (4.2) | 1 (6.7) |  |
| Number of re-operations (POD 0-30 days) |  |  |  |  |
| *0* | 39 (100.0) | 24 (100.0) | 15 (100.0) | - |
| Number of post-operative visits |  |  |  | 0.116 |
| *0* | 2 (5.1) | 0 (0.0) | 2 (13.3) |  |
| *1* | 28 (71.8) | 17 (70.8) | 11 (73.3) |  |
| *2* | 6 (15.4) | 5 (20.8) | 1 (6.7) |  |
| *3* | 2 (5.1) | 2 (8.3) | 0 (0.0) |  |
| *4* | 0 (0.0) | 0 (0.0) | 0 (0.0) |  |
| *5* | 1 (2.6) | 0 (0.0) | 1 (6.7) |  |
| Discharged from clinic after first visit | 27 (69.2) | 17 (70.8) | 10 (66.7) | 1.000 |

Abbreviations: IQR interquartile range, SSI surgical site infection, SD standard deviation, ED emergency department, POD post-operative day, ERCP retrograde cholangiopancreatography, EUS endoscopic ultrasound, IR interventional radiology.
^a^ Complications include: superficial and deep surgical site infections, biliary leak, and/or retained stone of sludge.


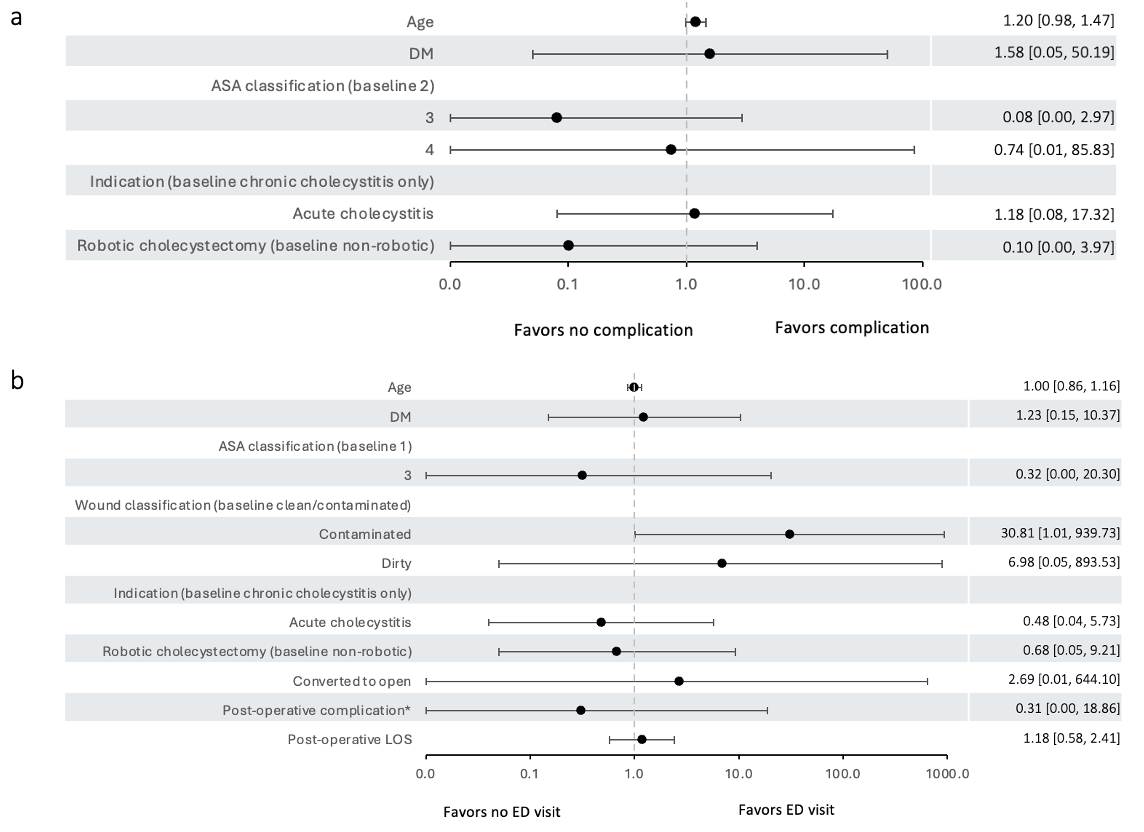
Figure 3. Multivariable analysis of complications (a), and 30-day emergency department visit (b) for percutaneous cholecystectomy subgroup. Thirty-day re-admission analysis not performed due to collinearity of multiple variables.
* Complications included: superficial and deep surgical site infections, biliary leak, and/or retained stone of sludge.

Table 13. Subgroup multivariable analysis of post-operative outcomes.

|  | **Acute cholecystitis subgroup** | | | | **Percutaneous cholecystostomy at time of surgery subgroup** | | | |
| --- | --- | --- | --- | --- | --- | --- | --- | --- |
| **Adjusted Outcome** | **RC, %** | **Non-RC, %** | **Odds ratio [95% CI]^b^** | **p-value** | **RC, %** | **Non-RC, %** | **Odds ratio [95% CI]^b^** | **p-value** |
| Complication^a^ | 4.8 | 10.3 | 0.59 [0.14, 2.42] | 0.468 | 4.2 | 20.0 | 0.10 [0.00, 3.97] | 0.222 |
| Re-admission^c^ | 7.7 | 7.5 | 0.80 [0.22, 2.91] | 0.729 | 0.0 | 26.7 | - | - |
| ED presentation^d^ | 22.1 | 27.1 | 1.02 [0.48, 2.18] | 0.964 | 20.8 | 33.3 | 0.68 [0.05, 9.21] | 0.772 |

## ^a^ Complications included: superficial and deep surgical site infections, biliary leak, retained stone of sludge. Outcomes reflected age, diabetes, ASA classification, surgical indication (in percutaneous cholecystostomy subgroup analysis only), percutaneous cholecystostomy tube (in acute cholecystitis subgroup analysis only), conversion to open (removed in percutaneous analysis due to collinearity); sex excluded due to collinearity; n=208 in acute cholecystitis analysis; n=39 in percutaneous cholecystostomy analysis.

^b^ Baseline non-robotic cholecystectomy.

^c^ Outcomes reflected age, sex, diabetes, ASA classification, wound classification, percutaneous cholecystostomy tube, conversion to open, post-operative complication, post-operative length of stay; re-admission analysis n=183 in acute cholecystitis subgroup.

^d^ Outcomes reflected age, sex (excluded in percutaneous cholecystectomy subgroup analysis due to collinearity), diabetes, ASA classification, wound classification, surgical indication (in percutaneous cholecystostomy subgroup analysis only), percutaneous cholecystostomy tube (in acute cholecystitis subgroup analysis only), conversion to open, post-operative complication, post-operative length of stay; n=207 in acute cholecystitis subgroup; n=34 in percutaneous cholecystostomy subgroup.
